# A Slow-Digesting Carbohydrate Diet during Rat Pregnancy Protects Offspring from Non-Alcoholic Fatty Liver Disease Risk through the Modulation of the Carbohydrate-Response Element and Sterol Regulatory Element Binding Proteins

**DOI:** 10.3390/nu11040844

**Published:** 2019-04-14

**Authors:** Rafael Salto, Manuel Manzano, María Dolores Girón, Ainara Cano, Azucena Castro, José Dámaso Vílchez, Elena Cabrera, José María López-Pedrosa

**Affiliations:** 1 Department of Biochemistry and Molecular Biology II, School of Pharmacy, University of Granada, Campus de Cartuja, 18071 Granada, Spain; rsalto@ugr.es (R.S.); e.damaso@go.ugr.es (J.D.V.); elenacc_20@hotmail.com (E.C.); 2 Abbott Nutrition R&D, Abbott Laboratories, 18004 Granada, Spain; manuel.manzano@abbott.com (M.M.); jose.m.lopez@abbott.com (J.M.L.-P.); 3 OWL Metabolomics, Parque Tecnológico de Bizkaia, 48160 Deiro, Spain; acano@owlmetabolomics.com (A.C.); acastro@owlmetabolomics.com (A.C.)

**Keywords:** early programming, hepatic lipogenesis, insulin-resistant pregnancy, metabolic flexibility, non-alcoholic fatty liver disease, slow digesting carbohydrates

## Abstract

High-fat (HF) and rapid digestive (RD) carbohydrate diets during pregnancy promote excessive adipogenesis in offspring. This effect can be corrected by diets with similar glycemic loads, but low rates of carbohydrate digestion. However, the effects of these diets on metabolic programming in the livers of offspring, and the liver metabolism contributions to adipogenesis, remain to be addressed. In this study, pregnant insulin-resistant rats were fed high-fat diets with similar glycemic loads but different rates of carbohydrate digestion, High Fat-Rapid Digestive (HF–RD) diet or High Fat-Slow Digestive (HF–SD) diet. Offspring were fed a standard diet for 10 weeks, and the impact of these diets on the metabolic and signaling pathways involved in liver fat synthesis and storage of offspring were analyzed, including liver lipidomics, glycogen and carbohydrate and lipid metabolism key enzymes and signaling pathways. Livers from animals whose mothers were fed an HF–RD diet showed higher saturated triacylglycerol deposits with lower carbon numbers and double bond contents compared with the HF–SD group. Moreover, the HF–RD group exhibited enhanced glucose transporter 2, pyruvate kinase (PK), acetyl coenzyme A carboxylase (ACC) and fatty acid (FA) synthase expression, and a decrease in pyruvate carboxylase (PyC) expression leading to an altered liver lipid profile. These parameters were normalized in the HF–SD group. The changes in lipogenic enzyme expression were parallel to changes in AktPKB phosphorylation status and nuclear expression in carbohydrate-response element and sterol regulatory element binding proteins. In conclusion, an HF–RD diet during pregnancy translates to changes in liver signaling and metabolic pathways in offspring, enhancing liver lipid storage and synthesis, and therefore non-alcoholic fatty liver disease (NAFLD) risk. These changes can be corrected by feeding an HF–SD diet during pregnancy.

## 1. Introduction

Humans have developed metabolic adaptations to promote energy storage in periods of fasting. They are especially suited to stimulating fat storage from other nutrients and carbohydrates as an evolutionary advantage for famine periods. However, alterations to this tightly regulated process have a relevant role in well-known pathological situations such as diabetes or obesity. It is also important during development, as catch-up growth in children after nourishment periods, or in the perinatal period, since it has been clearly established that a mother’s nutrition effects the metabolic performance of offspring in adulthood.

Metabolic flexibility is one of the main parameters that is modified by the glycemic index (GI) of dietary carbohydrates. Metabolic flexibility is the capability of an organism to select fuel oxidation in function of the nutrient availability (or the prediction that the organism makes about the nutrient availability). A relevant situation that involves fuel selection, fat storage and metabolic flexibility takes place during pregnancy [1]. Undernourishment or high-fat (HF) diets during this period lead to severe alterations in the offspring, both in human and animal models. In rats, feeding high-fat diets during pre-mating and/or gestation causes weight gain and glucose intolerance in offspring, regardless the post-weaning diet [2,3]. Therefore, dietary alterations during pregnancy can have a strong impact on the metabolic flexibility of the offspring in adulthood.

Dietary carbohydrates are able to promote metabolic adaptations to facilitate fat storage in a coordinated way, involving short term regulatory processes mainly involving changes in hormone (insulin and glucagon) secretion and response, as well as changes in signaling pathways (for example phosphorylation and regulation of key enzymes in response to the hormone secretion). Furthermore, carbohydrates are able to promote long-term adaptations in metabolism, involving modifications of gene expression at the cell nucleus that over time sustain the channeling of glucose to fat [4,5].

In a previous work [6], we have shown that the offspring of pregnant rats fed a high-fat diet containing slow-digesting (SD) carbohydrates during gestation (HF–SD group) seemed to protect against an increase in adipose tissue mass during adolescence. The HF–SD animals had reduced body fat mass and lower levels of cholesterol and triacylglycerols (TAGs) in plasma when compared with their counterparts, who were exposed to high-fat and rapidly digestible carbohydrate diets (HF–RD group) during gestation.

In this work, we have analyzed the effects of HF–SD and HF–RD diets during pregnancy in the liver metabolism of offspring in adolescence. Our results indicate that in the HF–RD group, liver metabolism is modified to promote glucose uptake that is converted into TAGs, producing changes in the lipid profile that indicate an enhanced risk of non-alcoholic fatty liver disease (NAFLD) in the offspring. On the contrary, the offspring from the HF–SD group showed a decreased tendency of lipogenesis and lipid storage. The results highlight the relevance of liver metabolic programming during pregnancy to control body homeostasis of the offspring. The supplementation of diet during pregnancy with slow-digestive carbohydrates normalizes metabolic and signaling pathways promoting metabolic flexibility, and appears to exert a protective effect on the offspring.

## 2. Materials and Methods

### 2.1. Animal and Housing

Female and male Sprague Dawley rats (aged 10 weeks) were purchased from Charles River Laboratories (Wilmington, MA, USA). Animals were maintained on a 12-h light/12-h dark cycle at 23–24 °C with food and water available ad libitum. All experimental procedures were approved by the Animal Welfare Committee at Estación Experimental del Zaidín-CSIC (Granada, Spain) in accordance with guidelines and recommendations of Spanish legislations on animal welfare and the European Convention for the Protection of Vertebrate Animals used for Experimental and other Scientific Purposes.

### 2.2. Diets and Experimental Design

**Figure 1:**
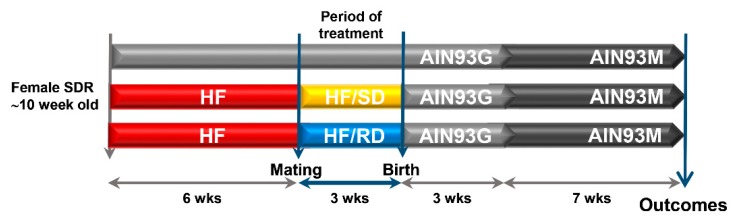
Experimental model. Virgin rats were assigned to one of three experimental groups: reference dams fed a standard rodent diet before mating and throughout pregnancy; dams fed a high-fat diet (HF) six weeks before mating and then an HF diet containing either carbohydrates with a high (HF–RD) or low (HF–SD) digestion rate throughout pregnancy. At delivery, all the animals were fed the standard rodent diet for the remainder of the study (10 weeks). SDR, Sprague-Dawley rats.

A detailed description of the experimental diets and design has been previously described [6]. In brief, a model of insulin resistance was used by feeding female rats with a highly palatable obesogenic lard diet (HF; 20.5% fat, 24.2% protein, 41.5% carbohydrates and 7.9% fiber per weight) prior to mating for six weeks. Control animals were fed a standard reference diet AIN93M. Rats were mated for three days and then randomly assigned to one of the experimental diets during gestation: a high-fat diet containing slow-digesting carbohydrates (HF–SD group), a high-fat diet containing rapid-digesting carbohydrates (HF–RD group) or an AIN93G diet for the reference group. Composition of the three diets used during gestation is described in Martin et al. [6] (Supplementary Table S1). All diets were prepared at Abbott Nutrition R&D facilities. At the delivery, all animals were fed AIN93G diets regardless of the diet consumed during pregnancy. On day 21 after delivery, all pups were weaned onto the AIN93M diet and housed for seven additional weeks (Figure 1). At the outcome, insulin and glucagon serum concentrations were assayed (Supplementary Table S2).

### 2.3. Liver Lipidomics Analysis

Absolute concentration of TAGs in the liver was measured using a triglycerides-LQ kit (Spinreact, Barcelona, Spain). Metabolite profiles were analyzed as previously described [7,8]. Briefly, two separate UHPLC-time-of-flight (TOF)–MS-based platforms (Agilent Technologies, Santa Clara, CA, USA) analyzing methanol and chloroform–methanol liver extracts were combined to semi-quantify lipid species. Non-esterified fatty acyls, bile acids and lysoglycerophospholipids were analyzed in the methanol extract platform. For this, methanol was added to the frozen liver tissue (30:1, *v*/*w*), and this mixture was homogenized with a Precellys 24 grinder (Precellys, Montigny-le-Bretonneux, France), followed by protein precipitation. The methanol used for extraction was spiked with internal standards not found in liver tissue using the same method. After brief vortex mixing, samples were incubated overnight at −20 °C. Supernatants were collected and dried after centrifugation at 16,000× *g* for 15 min. The dried extracts were resuspended in methanol, centrifuged at 16,000× *g* for 5 min and supernatants were collected and transferred to vials for UHPLC–MS (Agilent Technologies, Santa Clara, CA, USA) analysis.

The chloroform–methanol extract platform provided coverage over glycerolipids, glycerophospholipids, sterol lipids and sphingolipids. Liver tissues were homogenized in the Precellys 24 grinder by mixing with chloroform–methanol (2:1, *v*/*v*) and sodium chloride (50 mmol/L) (overall ratio 1:30:3, *w*/*v*/*v*), followed by protein precipitation. The extraction solvent was spiked with internal standards not detected using the same method. After brief vortex mixing, samples were incubated at −20 °C for 1 h. After centrifugation at 16,000× *g* for 15 min, the lower organic phase was collected and the solvent removed. The dried extracts were then resuspended in acetronitrile–isopropanol (1:1), centrifuged at 16,000× *g* for 5 min and, finally, supernatants were transferred to vials for UHPLC–MS analysis. Lipid nomenclature follows the LIPID MAPS convention, www.lipidmaps.org.

This combined analysis was established for rodent liver tissue by OWL Metabolomics (Derio, Spain). Data obtained with the UHPLC–MS were processed with the TargetLynx application manager for MassLynx 4.1 (Waters Corp., Milford, MA, USA). Intra- and inter-batch normalization followed the procedure published by Martinez-Herranz et al. [9]. All the calculations were performed with R v3.2.0 (R Development Core Team, Vienna, Austria, 2010).

### 2.4. Western Blot Analysis

Liver lysates were obtained in lysis buffer (RIPA buffer containing protease and phosphatase inhibitors). The lysate was centrifuged at 16,000× *g* at 4 °C for 15 min. The supernatant was transferred to a new microcentrifuge tube (1.5 mL) and sonicated for 15 s (cycle 0.5, amplitude 60%). Nuclear extracts from liver samples were prepared as described [10].

Protein concentration was determined using the bicinchoninic acid method, and 20–60 μg were loaded for western blot. The following antibodies were used: carbohydrate-responsive element-binding protein (ChREBP), glucose transporter 2 (GLUT2), sterol regulatory element-binding protein (SREBP) SREBP1 and SREBP2 from Santa Cruz Biotechnology (Santa Cruz, CA, USA). Acetyl Coenzyme A carboxylase (ACC), phosphoenolpyruvate carboxykinase (PEPCK), total and phospho-(Ser473)-PKBAkt, AMP-activated protein kinase α2 (AMPKα2) and phospho-AMPKα2 (Thr172), ERK1/2 and phospho-p44/42 Erk1/2 (Thr202/Tyr204), mammalian target of rapamycin (mTOR) and phospho-mTOR (Ser2448) were provided by Cell Signaling (Beverly, MA, USA). Pyruvate carboxylase (PyC) antibody was raised at Salto’s laboratory. GAPDH (Sigma-Aldrich, Saint Louis, MO, USA) was used as a load control. Data were normalized using the values of the reference animals as 100%.

### 2.5. Glycogen Content

Hepatic glycogen was isolated as described by Chang and Exon [11]. Liver homogenates (10%) were made in 30 mmol/L HCl and spread evenly on pieces of filter paper (Whatman 3M chromatography paper, 2.0 × 2.0 cm) in duplicate. The papers were dropped immediately into a beaker containing 66% ethanol and stirred gently by a rotating magnet screened from the papers by a wire mesh. The papers were subsequently washed three times for 40 min in 66% ethanol. Next, they were briefly rinsed with acetone and dried under a stream of warm air. The dried filter papers were cut into four pieces and placed in a tube containing 0.4 mL of 0.2 mol/L acetate buffer, pH 4.8; 0.2 mg of amylo-α-1,4-a-1,6-glucosidase; and H_2_O, to a final volume of 2 mL. The vials were incubated for 90 min at 37 °C with gentle shaking. Appropriate controls were prepared by incubating aliquots of homogenate in acetate buffer minus amyloglucosidase. Glucose concentration in the incubated samples was determined by the glucose oxidase method.

### 2.6. Statistical Analyses

One-way ANOVA test was applied when comparing three groups, and a Student’s *t* test was used when comparing two groups. Homoscedasticity was checked by Barlett’s test, and non-parametric tests were applied when appropriate. Differences were considered significant at *p* < 0.05.

## 3. Results

In this article, we have addressed the question of whether the changes owing to a high-fat/rapid-digestive carbohydrates diet during pregnancy produces metabolic adaptations in the liver of the offspring. This question is relevant, since the liver plays a central role in the metabolic flexibility of the organism and, therefore, is the key organ with the capacity to distribute nutrients to the rest of the organs and tissues. A second relevant question to be addressed was to determine if a slow-digesting carbohydrate diet during pregnancy could prevent and normalize the adverse effects of a high-fat diet on this organ in offspring.

**Figure 2:**
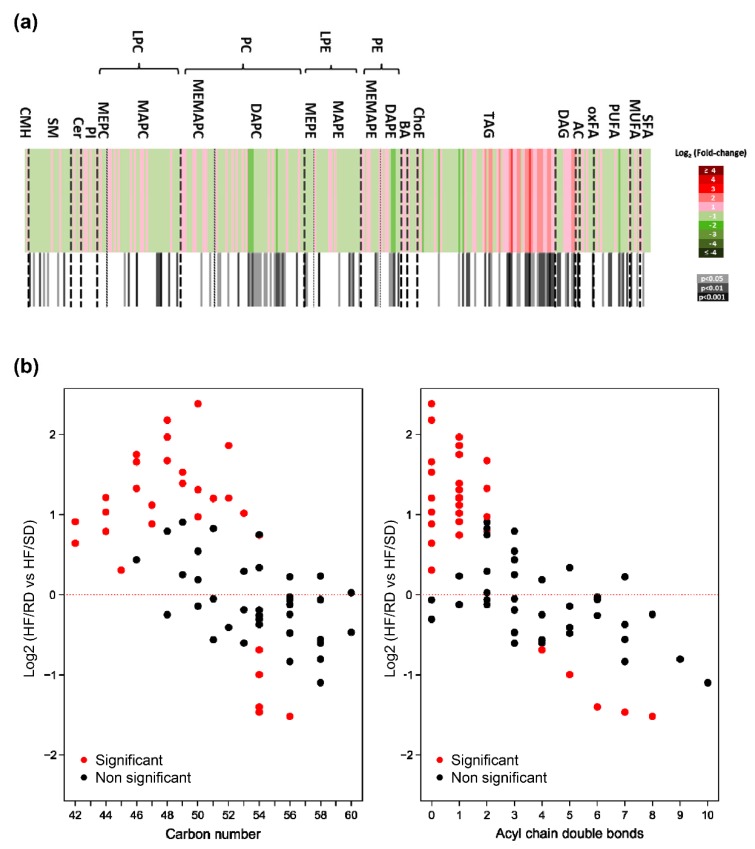
Rat liver lipidome. (**a**) Heat map showing relevant changes in liver lipids. Each metabolite is shown as a line whose color is defined by the sign and magnitude of the change. Adjacent column to each comparison shows the results of the *t* test (*p* value). Color scales are shown in the right: upper scale, Log2 fold-change; lower scale, statistical significance (*n* HF–SD = 7, *n* HF–RD = 8). BA, bile acids; Cer, ceramides; CMH, monohexosyl ceramide; ChoE, cholesterol esters; DAGs, diglycerides; DAPC, diacylglycerophosphocholine; DAPE, diacylglycerophosphoethanolamine; oxFA, oxidized free fatty acids; LPCs, lysophosphatidylcholines; LPEs, lysophosphatidylethanolamines; MEPC, 1-Monoetherglycerophosphocholine; MAPC, MonoacylglyceroPhosphocholine; MEMAPC, 1-ether, 2-acylglycerophosphocholine; PCs, phosphatidylcholines; PEs, phosphatidylethanolamines; PIs, phosphatidylinositols; MUFAs, monounsaturated fatty acids; PUFAs, polyunsaturated fatty acids; SFAs, saturated fatty acids; SMs, sphingomyelins; TAGs, triacylglycerols. (**b**) Correlation of HF–RD vs. HF–SD fold-change with TAG carbon number (left), and with acyl chain double bonds (right).

**Figure 3:**
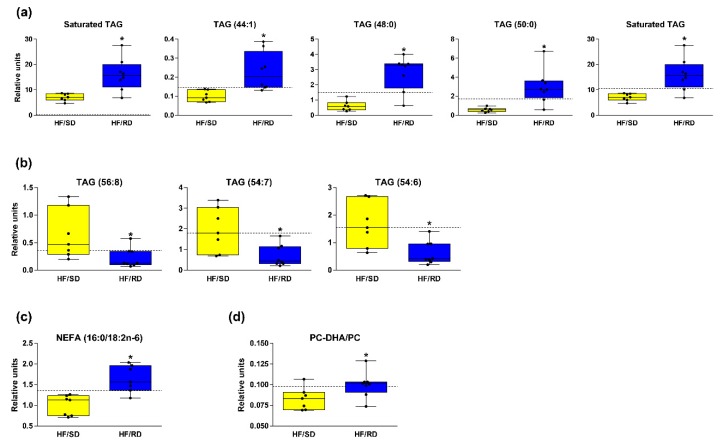
Relevant lipid species and ratios. (**a**) TAGs enriched in saturated fatty acids. (**b**) TAGs enriched in polyunsaturated fatty acids. (**c**) Lipogenic index. (**d**) Ratio Docosahexaenoyl Phosphatidylcholine/Phosphatidylcholine (PC–DHA/PC). The bar plots show the normalized values. The boxes range from 25%–75% percentiles; the 5% and 95% percentiles are indicated as error bars; single-data points are indicated by dots. Medians are indicated by horizontal lines within each box. Lines in all graphs show the reference group mean value. * p < 0.05 compared to the HF–SD group. NEFA, Non-esterified Fatyy Acids.

For this purpose, liver lipidome was first analyzed to depict long-term metabolic changes induced by the different diets during pregnancy on adolescent offspring (Figure 2), and 309 lipid metabolites were individually semi-quantified. Data were calibrated with quality controls, and changes in relative abundance of the liver lipidomes were searched, comparing the HF–RD and HF–SD experimental groups (Supplementary Table S3). Results are plotted as a heat map in Figure 2a. The liver lipid profile of the HF–RD animals was significantly different to those in the HF–SD, with significant differences (*p* < 0.05) in 99 lipid metabolites. The largest differences found were detected among TAGs; adolescent HF–RD rats had higher amounts of TAGs and diglycerides (DAGs) in their livers compared with the HF–SD animals. Besides, in the HF–RD group, TAGs had fatty acids (FAs) with lower carbon numbers and double-bond content than in the HF–SD animals, showing a characteristic pattern (Figure 3b).

As observed in Figure 2b, TAGs showed a remarkable profile in the livers of the HF–RD vs. HF–SD rats. TAG concentrations in the livers of the HF–RD presented a marked, gradual increase in the concentration of TAGs with shorter acyl chains and less unsaturation when compared with the HF–SD. The concentration of saturated or monounsaturated TAGs increased in the HF–RD when compared with the HF–SD, since the length of their carbon acyl chains was generally medium (16 carbons, palmitic acid). Some individual TAG species in the HF–RD were much higher than in the HF–SD, with TAGs (48:0) (TAGs (16:0/16:0/16:0)) and TAGs (50:0) (TAGs (16:0/16:0/18:0)) being the ones that changed the most (fold change 4.53 and 5.22, respectively) (Figure 3a). On the contrary, the concentration of long carbon acyl chain polyunsaturated TAGs decreased in the HF–RD when compared with the HF–SD (Figure 3b). The length of the carbon acyl chains in the HF–SD was 18 carbons or longer (Figure 3b). The HF–SD rats showed lower levels of TAG species enriched in saturated fatty acids (SFAs) than the reference group, and equal or higher levels of unsaturated TAG species than the same group.

A hallmark of NAFLD is the accumulation of liver TAGs, driven by increased palmitate and decreased polyunsaturated fatty acids (PUFAs) [7]. The ratio of FA (16:0/18:2n-6), called the lipogenic index [12], increased 63% in the HF–RD vs. HF–SD rats (Figure 3c).

The heat map also revealed a significant decrease in some phosphatidylcholine (PC), especially diacyl-PC, and phosphatidylethanolamine (PE) species in the HF–RD compared to the HF–SD group. Phosphatidylcholines (PCs) formed via phosphatidylethanolamine (PE) N-Methyltransferase (PEMT) pathway are primarily enriched in long-chain polyunsaturated fatty acids (PUFAs), such as docosahexaenoic acid (22:6n-3). Thus, the increased PC (22:6n-3) to total PC ratio (20%, *p* ≤ 0.05) found in HF–RD rats when compared with HF–SD rats (Figure 3d) suggests decreased PEMT flux in their livers, since previous studies indicated a correlation between the PC–DHA/PC ratio and the estimation of PEMT activity [8,13].

**Figure 4:**
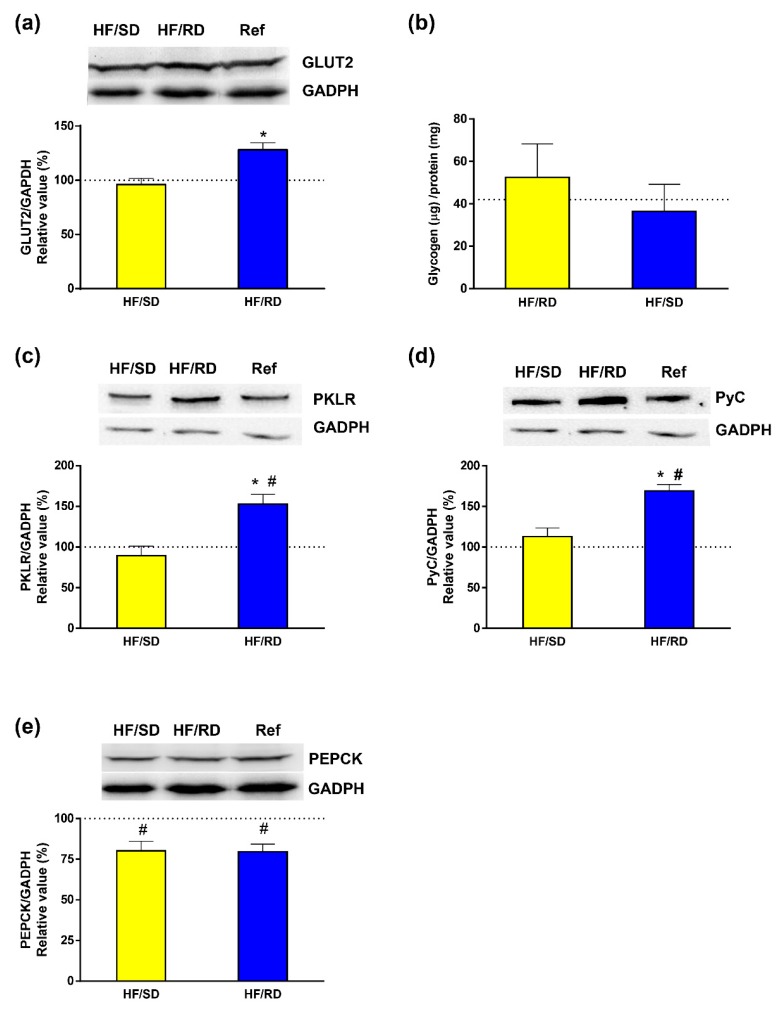
Liver carbohydrate metabolism. The expression of relevant transporters and enzymes has been analyzed by western blot; liver glycogen content has been assayed enzymatically. (**a**) GLUT2 transporter. (**b**) Glycogen content. (**c**) Liver pyruvate kinase isoenzyme (PKLR). (**d**) Pyruvate carboxylase (PyC). (**e**) Phosphoenol pyruvate carboxykinase (PEPCK). Results have been referred to reference rats (100% value). Results are mean ± SEM (*n* = 6 animals per group). * *p* < 0.05 compared to the HF–SD group; # *p* < 0.05 compared to the reference group.

**Figure 5:**
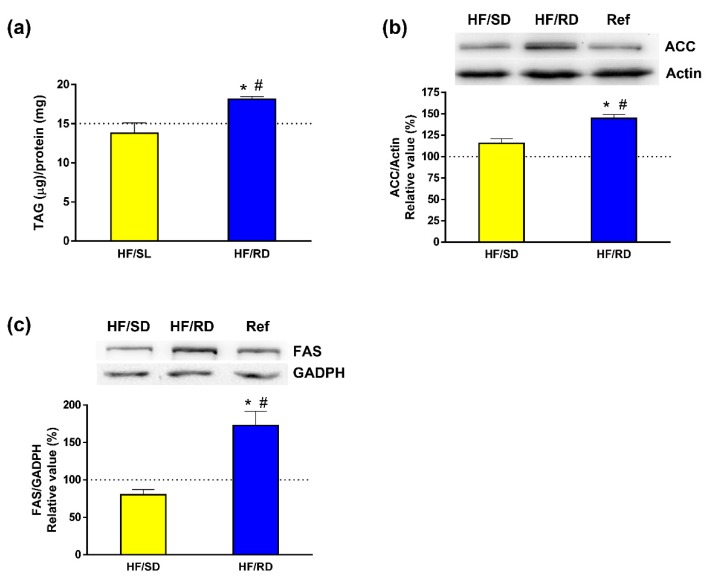
Liver lipogenesis. The expression of key enzymes has been analyzed by western blot, and liver triglyceride content has been assayed enzymatically. (**a**) Triacylglicerols (TAG) content. (**b**) Acetyl Coenzyme A carboxylase (AAC). (**c**) Fatty acid synthase (FAS). Results have been referred to control rats (100% value). Results are mean ± SEM (*n* = 6 animals per group). * *p* < 0.05 compared to the HF–SD group; # *p* < 0.05 compared to the reference group.

Next, we tried to elucidate if the changes observed in the lipid profile of the offspring could be related to modifications in glucose transporter expression and carbohydrate (Figure 4) and lipid metabolism (Figure 5) key enzymes. For that purpose, glucose transporter (GLUT2) was measured in the liver tissue of adolescent rats. Western blots of glyceraldehyde 3-phosphate dehydrogenase (GAPDH) were used as a load control. Our results showed that the HF–RD group had a significantly higher expression of GLUT2 compared to HF–SD rats, whose GLUT2 expression was similar to the reference group.

To address the fate of glucose in the liver of the HF–RD group, glycogen content and liver pyruvate kinase (PK) isoenzyme were determined as indicators of glycolytic flux. Glycogen content was similar in all experimental groups. In addition, glucokinase and glycogen phosphorylase activities were enzymatically assayed (Supplementary Figure S1) and no significant differences were found among experimental groups. Therefore, in the HF–RD group, the enhanced glucose uptake was not directed to glycogen, and we targeted a key enzyme of the glycolytic pathway, PK. Our results show that the expression of the PK main liver isoform (PKLR) was significantly higher in the HF–RD group with respect to the HF–SD and reference groups, having similar levels of both.

In addition, liver gluconeogenesis was analyzed in offspring by measuring the expression of pyruvate carboxylase (PyC) and phosphoenolpyruvate carboxykinase (PEPCK) (Figure 4d,e). Our results showed that the levels of PEPCK expression were similar between the two HF groups, and significantly lower compared with the reference group. More interestingly, expression of PyC in liver lysates was significantly higher in the HF–RD compared with the HF–SD and reference groups.

Next, the involvement of dietary intervention during pregnancy was analyzed in liver lipogenesis. The enzymatic measurement of total TAG content in the liver from the different experimental groups (Figure 5a) indicated that TAGs were being accumulated in the liver of the HF–RD when compared to the other two offspring groups. Furthermore, the expression of acetyl-CoA carboxylase (ACC) and fatty acid synthase (FAS), two key proteins involved in the metabolism of lipids in the liver, were measured. Both the levels of ACC (Figure 5b) and FAS (Figure 5c) were significantly higher in the HF–RD than in the HF–SD and reference groups.

**Figure 6:**
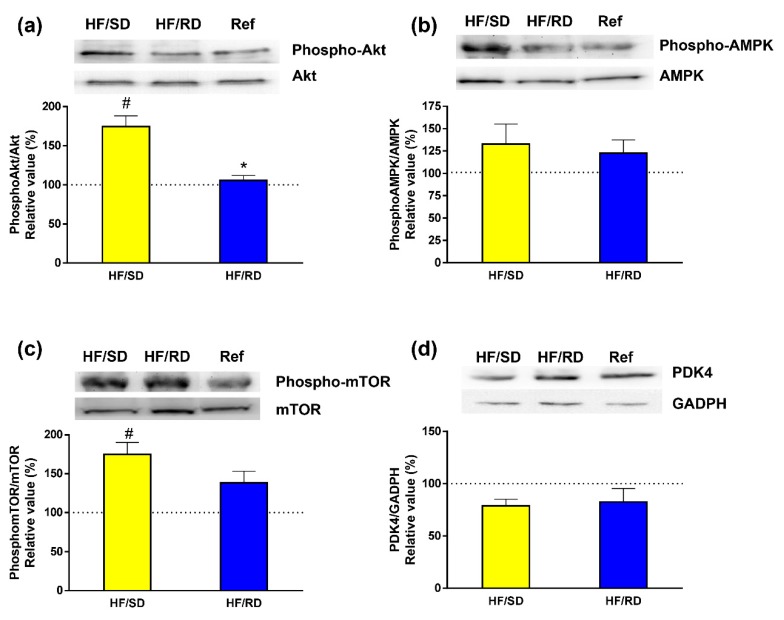
Signaling pathways involved in the regulation of liver metabolism. The phosphorylation of key kinases has been analyzed by western blot. (**a**) PKB–Akt, (**b**) AMPK, (**c**) mTOR and (**d**) PDK4. Results have been referred to control rats (100% value). Results are mean ± SEM (*n* = 6 animals per group). * *p* < 0.05 compared to the HF–SD group; # *p* < 0.05 compared to the reference group.

To study the effects of dietary carbohydrates as regulators of signaling pathways involved in glucidic and lipid homeostasis was our next task. Therefore, we studied the phosphorylation status of kinases that are essential for the regulation of these pathways (Figure 6). We analyzed the activation of PKBAkt (Figure 6a) as a marker of insulin responsiveness in the liver [4]. Our results indicate that in the HF–SD group there was a significant increase in the phosphorylation of the kinase compared with the HF–RD and reference groups.

Next, we assayed the activation of AMPK and mTOR, since they are involved in the control of cell growth and development [14,15]. While AMPK showed similar phosphorylation levels in all groups (Figure 6b), the phosphorylation of mTOR was higher in the HF–SD group compared with the other two groups, reaching significance with respect to the reference group.

Pyruvate dehydrogenase complex (PDC) constitutes one of the main decision points in the competition between fatty acids and glucose for oxidation [1]. The expression of PDK4 as the main isoform widely distributed of PDK was also measured, revealing that there were similar levels among groups.

**Figure 7:**
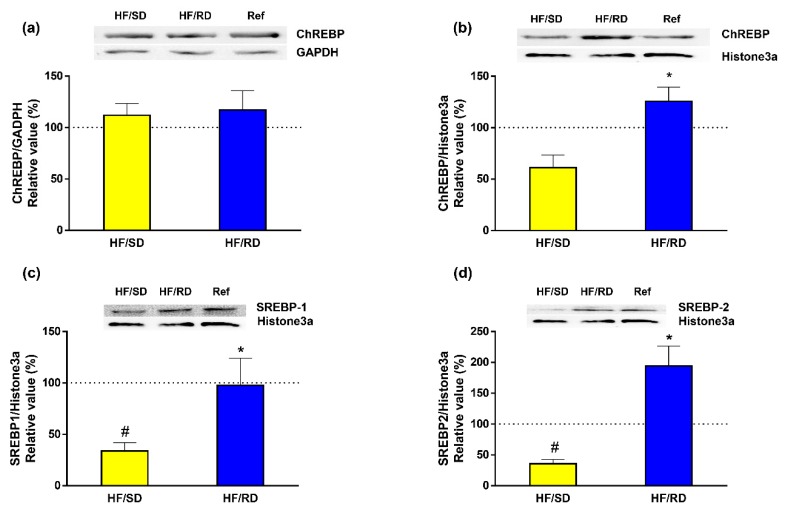
Carbohydrate-response element-binding protein (ChREBP) and sterol regulatory binding protein 1 and 2 (SREBP1 and SREBP2) expression in the liver. Total cellular (**a**) and nuclear (**b**) ChREBP content was analyzed by western blot. Nuclear expression of SREBP1 **(c)** and SREBP2 **(d)** was measured by western blot. Results were referred to rats (100% value). Results are mean ± SEM (*n* = 6 animals per group). * *p* < 0.05 compared to the HF–SD group; # *p* < 0.05 compared to reference group. Glyceraldehyde-3-phosphate Dehydrogenase, GADPH.

Finally, the expression in cell lysates and nuclear extracts of carbohydrate-response element-binding protein (ChREBP), which is regulated by dietary carbohydrates [16], was measured. Our results (Figure 7) indicate that, while the total amount of ChREBP protein was found to be similar in all experimental groups (Figure 7a), there was a significant increase in the nuclear concentration of the transcription factor in the HF–RD group (Figure 7b). Since the sterol regulatory binding protein 1 and 2 (SREBP1 and SREBP2) transcription factors act in a coordinated way with ChREBP to regulate transcription of lipogenic genes, the expression of these transcription factors was assayed in nuclear extracts from the different experimental groups (Figure 7c,d). The results indicate that SREBP1 and SREBP2 nuclear expression was significantly enhanced in the HF–RD group compared with the HF–SD group, while there was a decrease in the HF–SD group compared with the reference group.

## 4. Discussion

Avoiding pathologies such as obesity, diabetes, metabolic syndrome derived from the use of highly processed meals and sedentary life are objectives of the WHO. These diseases are associated with NAFLD, which is the commonest cause of chronic liver disease not only in adults but also in children, particularly in Western countries. NAFLD is a clinical term that refers to excess hepatic fat (5%–10% by weight) accumulation in the absence of excessive alcohol consumption [17]. Therefore, the promotion of healthy diets rich in fiber and slow-digesting carbohydrates could be of importance in the prevention of pathologies later in life [18].

Studies in animal models have been used to mimic human physiology, and the cafeteria diet (high-fat content and rapid-digesting carbohydrates) constitutes an appropriate model to test the importance of dietary intervention in the development of obesity, adiposity, metabolic syndrome and the associated NAFLD [19].

Dietary intervention has a direct influence on normal physiological function, but also in different pathological situations as metabolic syndrome, diabetes or cardiovascular disease [20]. Additionally, there are evidences that adverse situations during early development could have a direct impact in disease risk in later periods of life [21]. In fact, this is consistent with the theory of the developmental origins of health and disease (DOHaD) that environmental factors, including nutritional status, during the stages of development through the fetal and neonatal period are related to the risk of non-communicable diseases, such as metabolic syndrome, in later life [22].

One of the key points for dietary intervention is the control of glycaemia during pregnancy, especially among obese women since there is a well-established relationship between insulin resistance and adverse consequences for both mother and offspring. Studies are beginning to use different mixtures of carbohydrates in which rapid-digesting carbohydrates (sacharose) are replaced with low glycemic index (GI) carbohydrates as a rational approach [23].

It has been well established that a high-fat diet rich in rapid-digesting carbohydrates has deep effects on liver carbohydrate and lipid metabolism. However, the knowledge of molecular bases of how these changes can be translated to offspring is scarce. Some studies have reported that high-fat diets during the period of pre-mating or gestation induce glucose intolerance in pups, and this change is independent of the post-weaning diet [2,3]. Other studies have described that when high-fiber and high-protein diets are consumed during pregnancy and lactation, there is a regulation of satiety hormones and genes involved in glucose metabolism in offspring [5]. Furthermore, our previous results indicate that a high-fat, rapid-digestive carbohydrate diet during pregnancy promotes an increase in adipose tissue mass and a concomitant alteration of carbohydrate and lipid metabolism in this tissue in the offspring [6]. More interesting, these adipose tissue alterations can be corrected when slow digestive ones substitute the rapid-digesting carbohydrates.

In an individual, a high-fat diet containing rapid-digestive carbohydrates is able to increase liver glucose uptake, glycolysis and alter glycogen metabolism. At the same time, it inhibits gluconeogenesis. Therefore, under these circumstances the liver is a net glucose consumer, rather than a glucose exporter. If we pay attention to lipid metabolism, a diet enriched in rapid-digesting carbohydrates blocks the use of fatty acids to obtain energy and enhances the conversion of glucose to TAGs. Consequently, these diets promote a hepatic lipogenic program that has local and systemic consequences, such as an increase in blood TAGs and cholesterol, enhanced TAG transport to adipose tissue and, even worse, hepatic steatosis [17].

The question that this article addresses is to confirm if these alterations leading to NAFLD are also mimicked in the offspring of animals fed these diets during pregnancy [24], and whether a slow-digestive carbohydrate supplementation to pregnant mothers has any protective effect later on the offspring to prevent this risk.

In the HF–SD diet, the sugars (sucrose and maltodextrin) as rapidly metabolized carbohydrates were substituted for a mixture of slow-digestive carbohydrates composed of isomaltulose, resistant maltodextrins and fructooligosaccharides [6]. This dietary intervention during pregnancy has been proven to prevent excess adipogenesis in offspring by modulating adipose tissue metabolism. More important is to determine if these positive effects on adipose tissue are also present in the livers of the HF–SD offspring.

The liver has a key role on metabolism homeostasis, and it has been demonstrated that maternal obesity imposes a developmental programming on offspring, affecting mainly the liver, and pre-disposing to obesity and NAFLD [17,25].

Our results confirm that a high-fat diet containing rapid-digestive carbohydrates during pregnancy promotes a predisposition to develop NAFLD in the livers of offspring. These results are in agreement with previous studies [24,25].

The liver lipid profile of the HF–RD animals was significantly different from the HF–SD rats, mainly among TAGs. Remarkably, TAG and DAG concentrations in the livers of the HF–RD adolescent rats increased, with the acyl chains in TAGs being shorter and less unsaturated than in the HF–SD (Figure 3b). It should be highlighted that some individual TAG species in the HF–RD were greatly higher than those of the other groups (Figure 3a). Interestingly, Petry et al. [26] suggest that fetal imprinted genes may influence maternal circulating clinically relevant TAG concentrations early in pregnancy. Concretely, TAGs (44:1), which increased 2.32-fold in the livers of the HF–RD when compared with the HF–SD rats in our study (Figure 3a), were found to be very strongly associated to maternal and paternal transmissions and linked to insulin resistance.

Additionally, the concentration of polyunsaturated TAGs with long carbon acyl chains decreased in the HF–RD when compared with the HF–SD (Figure 3b), with the length of their carbon acyl chains being 18 carbons or longer (Figure 3b). Rhee et al. [4] found that lipids, especially serum TAGs of lower carbon numbers and double-bond content, were associated with an increased risk of diabetes, whereas lipids of higher carbon numbers and double-bond content were associated with a decreased risk.

There are data in the lipid profile that point out the possibility NAFLD developing in the HF–RD offspring. The more important results related with this hypothesis mean an increase in liver TAGs and lipogenic index combined with a significant decrease in PCs, and are considered as a hallmark of this pathology [27]. The decrease in PCs may affect the PC–DHA levels that are incorporated in very-low-density lipoproteins (VLDLs) secreted by the liver and transported to peripheral tissues. PCs are required for the assembly/secretion of lipoproteins [28,29]. Formerly, reduced PC levels would be expected to account for at least some of the TAG accumulation in the liver of HF–RD rats due to the reduced secretion of VLDLs from the liver [30].

In conclusion, our lipidomic study indicates that offspring from animals fed an HF–RD diet during pregnancy showed a lipid profile compatible with the early stages of liver steatosis. On the contrary, the supplementation of high-fat diets with slow-digestive carbohydrates during pregnancy had a protective effect on offspring, decreasing the risk of NAFLD. However, no liver histological changes were observed in the different experimental groups (data not shown), probably due to the short period of time (13 weeks after delivery) before the analysis. 

The observed changes in the lipidome with a clear effect of the HF–RD diet increasing the TAG content in liver could be explained by changes in the flux from glucose to lipids. Firstly, liver carbohydrate metabolism was studied by measuring the expression of GLUT2 transporter and the PK liver isoenzyme. Our results showed that the expression of GLUT2 was significantly higher in the HF–RD group with respect to the HF–SD and reference groups. GLUT2 is the main glucose and fructose transporter in the liver, which allows large bidirectional fluxes of glucose in and out the cell due to its low affinity and high capacity (high Vmax and Km for glucose) [31]. Therefore, increased expression of GLUT2 in the offspring from the HF–RD group indicates a permanent metabolic adaptation that, by the increase in liver glucose uptake, resembles a situation of hampered insulin response [32]. The increase in GLUT2 did not translated into a higher storage of glycogen in the HF–RD group. This result indicates that, in our experimental setting, dietary treatment during pregnancy did not alter liver glycogen storage and metabolism.

Pyruvate kinase is a glycolytic enzyme that catalyzes the last step, converting phosphoenolpyruvate to pyruvate. Pyruvate kinase exists in a number of isoforms, the liver type (PKLR) being the most abundant in that organ [33]. The combined higher expression of GLUT2 and PKLR in the HF–RD group points to increased glycolytic flux in these animals compared with the reference and HF–SD groups. 

The increase in glycolytic flux due to high glycemic index diets is usually combined with an inhibition of liver gluconeogenesis. The decrease of PEPCK in both groups of rats with respect to the reference group supports the inhibition of the gluconeogenic pathway. More interesting, liver PyC was significantly higher in the HF–RD compared with the HF–SD and control groups. This is a remarkable finding since PyC, in addition to its role in gluconeogenesis, is responsible for the anaplerotic replenishment of the Krebs cycle. This replenishment is needed in processes where Krebs cycle intermediaries are used for synthetic purposes, as in lipogenesis from glucose. Therefore, these results are a first insight that in the HF–RD group there was a permanent adaptation to promote lipid synthesis as the ultimate fate of glucose in liver. On the contrary, this channeling was corrected in the HF–SD group.

Up to this point, our liver results, lipid data and higher PyC expression put forward an active conversion of glucose to TAGs in the HF–RD group. Moreover, these animals had higher circulating TAGs and an increase in adipose mass [6]. Liver TAG content was higher in the HF–RD than in the other two groups, in agreement with the results on lipidomics, and corroborating the enhanced conversion of glucose into TAGs in this group. ACC and FAS that catalyze de novo synthesis of fatty acids in liver were significantly higher in the HF–RD than in the HF–SD and reference groups. ACC catalyzes the carboxylation of acetyl-CoA to Malonyl-CoA, the first committed step in long-chain fatty acid synthesis [34]. Later, these fatty acids will be incorporated into TAGs and/or phospholipids. Its expression is regulated at several levels, synthesis of the protein being one of them [35].

In the liver, FAS has long been categorized as a housekeeping protein, producing fat for the storage of energy when nutrients are present in excess. Fatty acids are packed into VLDL particles and delivered to adipose tissue and other extrahepatic tissues through the bloodstream [36]. Therefore, the overexpression of FAS in the HF–RD group, combined with reduced PC levels, explains the higher circulating TAGs observed in these animals [6] together with the increased deposits of TAGs on the liver. On the contrary, the overall analysis of the HF–SD group indicates that the carbohydrate composition of this diet mediates a metabolic state in which a lower use of glucose by glycolysis is combined with a decrease in the synthesis of fatty acids and TAGs.

In addition to regulating the expression of a variety of proteins, dietary carbohydrates also modulate signaling pathways involved in glucidic and lipidic homeostasis as Akt-, AMPK- or mTOR-dependent transduction routes. Insulin stimulates hepatic lipogenesis after feeding through an activation of the PI3-kinase–Akt pathway. Moreover, insulin fuels glycolysis, thus increasing the availability of lipogenic precursors [37,38]. However, high-fat diets are clearly associated with insulin resistance. The HF–SD diet significantly increased the phosphorylation of PKB–Akt, which was measured as a marker of insulin responsiveness in the liver compared with the HF–RD and reference groups. This result could point to better insulin sensitivity in the HF–SD group, which would be in agreement with the data from the postprandial glycemic peak and total glycemic response that were lower in HF–SD rats when compared to the HF–RD [6].

AMPK and mTOR are the main kinases responsible for integrating signals that control growth and development [14,15]. In a favorable energy status, mTOR is active, but when there is a fuel deficiency, activation of AMPK inhibits mTOR signaling [15]. Thus, the availability of nutrients leads to an activation of TOR protein kinase activity, while a depletion of amino acids or glucose points to an attenuation of mTOR phosphorylation [39]. AMPK, a sensor of fuel availability [40], showed similar phosphorylation levels in all groups. On the contrary, the phosphorylation of mTOR was higher in the HF–SD group compared with the other two groups. The regulation of mTOR is controlled by upstream signaling pathways, such as the insulin-dependent signaling pathway, and measured by the phosphorylation status of Akt–PKB and AMPK phosphorylation as a sensor of fuel availability [40]. Therefore, it could be concluded from these results that the higher phosphorylation of mTOR in the HF–SD rats was driven by the activation of the insulin pathway, rather than changes in AMPK activity.

Metabolic flexibility is the capability of a system to regulate fuel (primarily glucose and fatty acids) oxidation in response to nutrient availability. Thus, the possibility to change a substrate catabolism nutritional state depends on the steadiness between oxidation and storage capacities. PDC represents one of the key regulating points in the decision of the cell to use fatty acids or glucose as a fuel. This complex is usually active in tissues in the fed state, but the inhibition of its activity by pyruvate dehydrogenase kinase (PDK) is critical to maintaining energy homeostasis under nutritional conditions [1,41]. PDK isozymes phosphorylate specific serine residues in PDC [42]. Of all the known isozymes, PDK4 is highly expressed in the liver [1,41]. At the same time, PDK4 also controls glycolytic flux, since inactivation of PDC by PDKs blocks acetyl-CoA synthesis from pyruvate, resulting in a shift of pyruvate to the TCA cycle. Since PDK4 expression was similar among groups, it might demonstrate that the effects of the dietary intervention during pregnancy do not translate in a short-term regulation of metabolic flexibility in the liver, where PDK4 is relevant, but maybe involve long-term metabolic regulation of the metabolism at the gene-expression level.

The long-term regulation of metabolism induced by high GI diets is mediated by specific transcription factors that can bind to DNA and mediate the transcription of genes that code for the lipogenic enzymes. A relevant transcription factor is ChREBP, carbohydrate-response element-binding protein, which is activated by glucose-6-phosphate. ChREBP includes in its structure several phosphorylation sites and a glucose-sensing domain. In a low-GI diet or fasting, phosphorylation of ChREBP mediated by cyclic AMP keeps the transcription factor inactive at the cytosol. On the contrary, a high-GI diet increases xylulose-5-phosphate levels, which act as potent activators of the phosphatase A2. In the non-phosphorylated state, ChREBP migrates to the nucleus, and glucose-6-phosphate binds to the glucose-sensing domain. In this state, ChREBP enhances the transcription of the genes involved in the conversion of glucose to fat [16].

Our results indicate that, although the total amount of ChREBP protein (Figure 7) was similar in all experimental groups (Figure 7a), its nuclear concentration was increased in the HF–RD group. This finding could be due to an increase in xylulose-5-phosphate levels in these animals in response to the enhanced glucose uptake mediated by GLUT2. This event would activate phosphatase A2 and nuclear translocation of ChREBP. Furthermore, the increased nuclear levels of ChREBP in the HF–RD group are sufficient to explain the long-term modifications in the levels of key enzymes and transporters leading to a metabolic inflexibility that channels liver glucose to lipid storage, since GLUT2, PKLR, ACC and FAS expression is regulated at the transcription level by this transcription factor.

Transcription of the lipogenic genes is driven in a coordinated way with ChREBP by SREBP1 and SREBP2 transcription factors. These transcription factors have a complex regulation that involves not only a proteolytic activation, but also a regulation by carbohydrates [43]. Our results indicate that there is a synergic regulation through dietary intervention in the liver’s nuclear amount of SREBPs. Both transcriptional factors were incremented in the HF–RD group compared with the HF–SD group, and the levels of SRBEPs in the HF–SD group were also significantly lower compared with the reference group. 

## 5. Conclusions

The results presented in this article reinforce the importance of maternal nutrition during the early critical period of development, and suggest that offspring exposed to a maternal HF diet with rapid-digestible carbohydrates have a predisposition to develop NAFLD in later life. In contrast, these negative effects are ameliorated by a maternal HF diet with slow-digestible carbohydrates, probably through an increase in the sensitivity of the insulin signaling pathway, the modulation of key enzymes and transporters and the normalization of the lipid profile. This demonstrates the role carbohydrates play in a maternal diet on the developmental programming of metabolic flexibility in offspring, as well as the relevance of the liver in these adaptive processes.

## Supplementary Materials

The following are available online at https://www.mdpi.com/2072-6643/11/4/844/s1, Figure S1: (**a**) Glucokinase activity was measured in liver homogenates as described (Massa, L.; Baltrusch, S.; Okar, D.A.; Lange, A.J.; Lenzen, S.; Tiedge, M., Interaction of 6-phosphofructo-2-kinase/fructose-2, 6-bisphosphatase (PFK-2/FBPase-2) with glucokinase activates glucose phosphorylation and glucose metabolism in insulin-producing cells. Diabetes 2004, 53, 1020–1029). (**b**) Glycogen Phosphorylase b activity was measured spectrophotometrically as described in Board, M.; Hadwen, M.; Johnson, L. N., Effects of novel analogues of D-glucose on glycogen phosphorylase activities in crude extracts of liver and skeletal muscle. Eur. J. Biochem. 1995, 228, 753–761. Results are mean ± SEM (*n* = 6 animals per group). No significant differences were found among experimental groups; Table S1: Experimental diets during rat pregnancy; Table S2: Glucose, insulin and glucagon levels in fasted serum samples from the different experimental groups (*n* = 8); Table S3: Heat map showing relevant changes in liver lipids.

## References

[1] Zhang S., Hulver M.W., McMillan R.P., Cline M.A., Gilbert E.R. (2014). The pivotal role of pyruvate dehydrogenase kinases in metabolic flexibility. Nutr. Metab..

[2] Nivoit P., Morens C., Van Assche F.A., Jansen E., Poston L., Remacle C., Reusens B. (2009). Established diet-induced obesity in female rats leads to offspring hyperphagia, adiposity and insulin resistance. Diabetologia.

[3] Chen H., Simar D., Morris M.J. (2009). Hypothalamic neuroendocrine circuitry is programmed by maternal obesity: Interaction with postnatal nutritional environment. PLoS ONE.

[4] Rhee E.P., Cheng S., Larson M.G., Walford G.A., Lewis G.D., McCabe E., Yang E., Farrell L., Fox C.S., O’Donnell C.J. (2011). Lipid profiling identifies a triacylglycerol signature of insulin resistance and improves diabetes prediction in humans. J. Clin. Investig..

[5] Maurer A.D., Reimer R.A. (2011). Maternal consumption of high-prebiotic fibre or -protein diets during pregnancy and lactation differentially influences satiety hormones and expression of genes involved in glucose and lipid metabolism in offspring in rats. Br. J. Nutr..

[6] Martin M.J., Manzano M., Bueno-Vargas P., Rueda R., Salto R., Giron M.D., Vilchez J.D., Cabrera E., Cano A., Castro A. (2018). Feeding a slowly digestible carbohydrate diet during pregnancy of insulin-resistant rats prevents the excess of adipogenesis in their offspring. J. Nutr. Biochem..

[7] Barr J., Caballeria J., Martinez-Arranz I., Dominguez-Diez A., Alonso C., Muntane J., Perez-Cormenzana M., Garcia-Monzon C., Mayo R., Martin-Duce A. (2012). Obesity-dependent metabolic signatures associated with nonalcoholic fatty liver disease progression. J. Proteome Res..

[8] Martinez-Una M., Varela-Rey M., Cano A., Fernandez-Ares L., Beraza N., Aurrekoetxea I., Martinez-Arranz I., Garcia-Rodriguez J.L., Buque X., Mestre D. (2013). Excess S-adenosylmethionine reroutes phosphatidylethanolamine towards phosphatidylcholine and triglyceride synthesis. Hepatology.

[9] Martinez-Arranz I., Mayo R., Perez-Cormenzana M., Minchole I., Salazar L., Alonso C., Mato J.M. (2015). Enhancing metabolomics research through data mining. J. Proteom..

[10] Giron M.D., Sevillano N., Vargas A.M., Dominguez J., Guinovart J.J., Salto R. (2008). The glucose-lowering agent sodium tungstate increases the levels and translocation of GLUT4 in L6 myotubes through a mechanism associated with ERK1/2 and MEF2D. Diabetologia.

[11] Chan T.M., Exton J.H. (1976). A rapid method for the determination of glycogen content and radioactivity in small quantities of tissue or isolated hepatocytes. Anal. Biochem..

[12] Hudgins L.C., Hellerstein M., Seidman C., Neese R., Diakun J., Hirsch J. (1996). Human fatty acid synthesis is stimulated by a eucaloric low fat, high carbohydrate diet. J. Clin. Investig..

[13] Barbier-Torres L., Delgado T.C., Garcia-Rodriguez J.L., Zubiete-Franco I., Fernandez-Ramos D., Buque X., Cano A., Gutierrez-de Juan V., Fernandez-Dominguez I., Lopitz-Otsoa F. (2015). Stabilization of LKB1 and Akt by neddylation regulates energy metabolism in liver cancer. Oncotarget.

[14] Howell J.J., Manning B.D. (2011). mTOR couples cellular nutrient sensing to organismal metabolic homeostasis. Trends Endocrinol. Metab..

[15] Xu J., Ji J., Yan X.H. (2012). Cross-talk between AMPK and mTOR in regulating energy balance. Crit. Rev. Food Sci. Nutr..

[16] Uyeda K., Repa J.J. (2006). Carbohydrate response element binding protein, ChREBP, a transcription factor coupling hepatic glucose utilization and lipid synthesis. Cell Metab..

[17] Brumbaugh D.E., Friedman J.E. (2014). Developmental origins of nonalcoholic fatty liver disease. Pediatr. Res..

[18] Popkin B.M. (2006). Global nutrition dynamics: The world is shifting rapidly toward a diet linked with noncommunicable diseases. Am. J. Clin. Nutr..

[19] Sampey B.P., Vanhoose A.M., Winfield H.M., Freemerman A.J., Muehlbauer M.J., Fueger P.T., Newgard C.B., Makowski L. (2011). Cafeteria diet is a robust model of human metabolic syndrome with liver and adipose inflammation: Comparison to high-fat diet. Obesity.

[20] Joy T., Lahiry P., Pollex R.L., Hegele R.A. (2008). Genetics of metabolic syndrome. Curr. Diabetes Rep..

[21] Godfrey K.M., Lillycrop K.A., Burdge G.C., Gluckman P.D., Hanson M.A. (2007). Epigenetic mechanisms and the mismatch concept of the developmental origins of health and disease. Pediatr. Res..

[22] Heindel J.J., Vandenberg L.N. (2015). Developmental origins of health and disease: A paradigm for understanding disease cause and prevention. Curr. Opin. Pediatr..

[23] Clapp J.F. (2002). Maternal carbohydrate intake and pregnancy outcome. Proc. Nutr. Soc..

[24] Pereira T.J., Fonseca M.A., Campbell K.E., Moyce B.L., Cole L.K., Hatch G.M., Doucette C.A., Klein J., Aliani M., Dolinsky V.W. (2015). Maternal obesity characterized by gestational diabetes increases the susceptibility of rat offspring to hepatic steatosis via a disrupted liver metabolome. J. Physiol..

[25] Oben J.A., Mouralidarane A., Samuelsson A.M., Matthews P.J., Morgan M.L., McKee C., Soeda J., Fernandez-Twinn D.S., Martin-Gronert M.S., Ozanne S.E. (2010). Maternal obesity during pregnancy and lactation programs the development of offspring non-alcoholic fatty liver disease in mice. J. Hepatol..

[26] Petry C.J., Koulman A., Lu L., Jenkins B., Furse S., Prentice P., Matthews L., Hughes I.A., Acerini C.L., Ong K.K. (2018). Associations between the maternal circulating lipid profile in pregnancy and fetal imprinted gene alleles: A cohort study. Reprod. Biol. Endocrinol..

[27] Puri P., Baillie R.A., Wiest M.M., Mirshahi F., Choudhury J., Cheung O., Sargeant C., Contos M.J., Sanyal A.J. (2007). A lipidomic analysis of nonalcoholic fatty liver disease. Hepatology.

[28] West A.A., Yan J., Jiang X., Perry C.A., Innis S.M., Caudill M.A. (2013). Choline intake influences phosphatidylcholine DHA enrichment in nonpregnant women but not in pregnant women in the third trimester. Am. J. Clin. Nutr..

[29] Sherriff J.L., O’Sullivan T.A., Properzi C., Oddo J.L., Adams L.A. (2016). Choline, Its Potential Role in Nonalcoholic Fatty Liver Disease, and the Case for Human and Bacterial Genes. Adv. Nutr..

[30] Cano A., Alonso C. (2014). Deciphering non-alcoholic fatty liver disease through metabolomics. Biochem. Soc. Trans..

[31] Leturque A., Brot-Laroche E., Le Gall M. (2009). GLUT2 mutations, translocation, and receptor function in diet sugar managing. Am. J. Physiol. Endocrinol. Metab..

[32] Marks J., Carvou N.J., Debnam E.S., Srai S.K., Unwin R.J. (2003). Diabetes increases facilitative glucose uptake and GLUT2 expression at the rat proximal tubule brush border membrane. J. Physiol..

[33] Han S.M., Namkoong C., Jang P.G., Park I.S., Hong S.W., Katakami H., Chun S., Kim S.W., Park J.Y., Lee K.U. (2005). Hypothalamic AMP-activated protein kinase mediates counter-regulatory responses to hypoglycaemia in rats. Diabetologia.

[34] Tong L. (2005). Acetyl-coenzyme A carboxylase: Crucial metabolic enzyme and attractive target for drug discovery. Cell. Mol. Life Sci..

[35] Brownsey R.W., Boone A.N., Elliott J.E., Kulpa J.E., Lee W.M. (2006). Regulation of acetyl-CoA carboxylase. Biochem. Soc. Trans..

[36] Eissing L., Scherer T., Todter K., Knippschild U., Greve J.W., Buurman W.A., Pinnschmidt H.O., Rensen S.S., Wolf A.M., Bartelt A. (2013). De novo lipogenesis in human fat and liver is linked to ChREBP-beta and metabolic health. Nat. Commun..

[37] Jensen-Urstad A.P., Semenkovich C.F. (2012). Fatty acid synthase and liver triglyceride metabolism: Housekeeper or messenger?. Biochim. Biophys. Acta.

[38] Rui L. (2014). Energy metabolism in the liver. Compr. Physiol..

[39] Sengupta S., Peterson T.R., Sabatini D.M. (2010). Regulation of the mTOR complex 1 pathway by nutrients, growth factors, and stress. Mol. Cell.

[40] Huang J., Manning B.D. (2008). The TSC1-TSC2 complex: A molecular switchboard controlling cell growth. Biochem. J..

[41] Parillo M., Licenziati M.R., Vacca M., de Marco D., Iannuzzi A. (2012). Metabolic changes after a hypocaloric, low-glycemic-index diet in obese children. J. Endocrinol. Investig..

[42] Sugden M.C., Holness M.J. (2006). Mechanisms underlying regulation of the expression and activities of the mammalian pyruvate dehydrogenase kinases. Arch. Physiol. Biochem..

[43] Osborne T.F. (2000). Sterol regulatory element-binding proteins (SREBPs): Key regulators of nutritional homeostasis and insulin action. J. Biol. Chem..

