# Supplementary material for: A Slow-Digesting Carbohydrate Diet during Rat Pregnancy Protects Offspring from Non-Alcoholic Fatty Liver Disease Risk through the Modulation of the Carbohydrate-Response Element and Sterol Regulatory Element Binding Proteins
Source: Nutrients. 2019 Apr 14;11(4):844. doi: 10.3390/nu11040844 (PMC6521114; doi:10.3390/nu11040844)
Supplement: Supplementary file 1 [file nutrients-11-00844-s001.zip › nutrients-454319-supplememtary/Supplemental Figures.pdf]

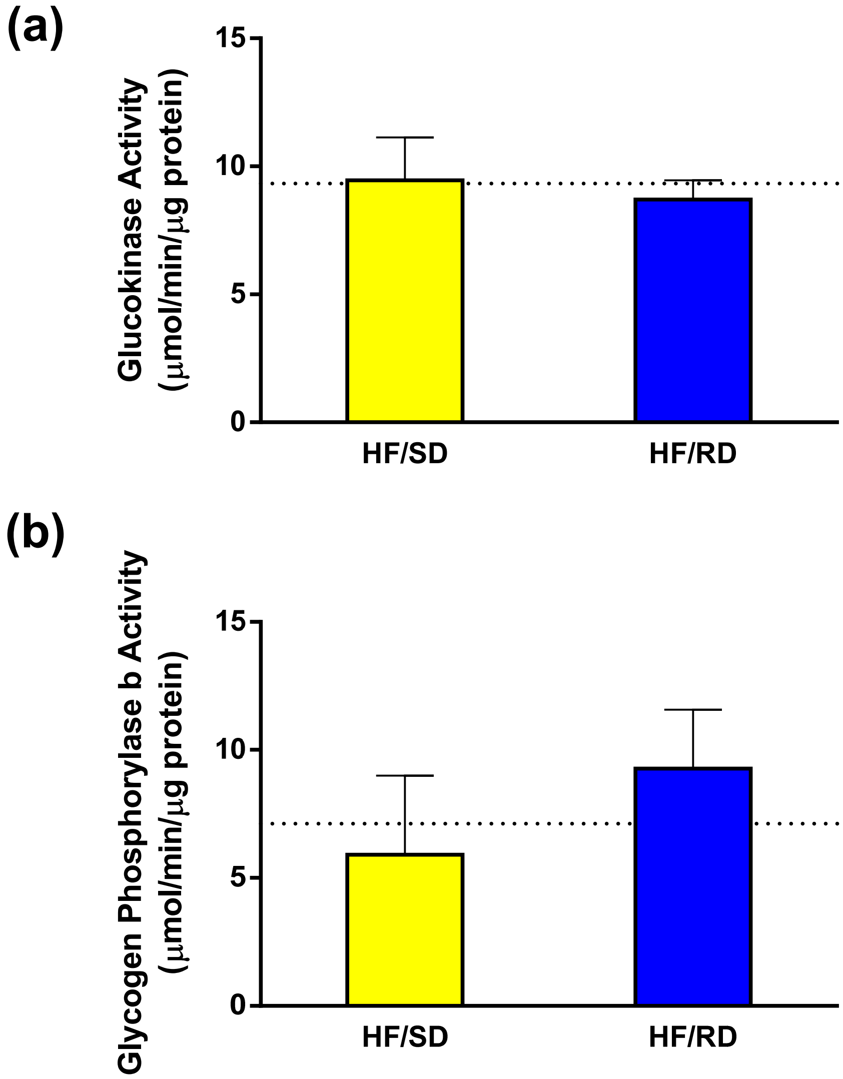

Supplementary Figure S1. (a) Glucokinase activity was measured in liver homogenates as described (Massa, L.; Baltrusch, S.; Okar, D. A.; Lange, A. J.; Lenzen, S.; Tiedge, M., Interaction of 6-phosphofructo-2-kinase/fructose-2, 6-bisphosphatase (PFK-2/FBPase-2) with glucokinase activates glucose phosphorylation and glucose metabolism in insulin-producing cells. *Diabetes* **2004,** 53, 1020-1029). **(b)** Glycogen Phosphorylase b activity was measured spectrophotometrically as described in Board, M.; Hadwen, M.; Johnson, L. N., Effects of novel analogues of D-glucose on glycogen phosphorylase activities in crude extracts of liver and skeletal muscle. *Eur. J. Biochem.* **1995**, 228, 753-761. Results are mean ± SEM (n=6 animals per group). No significant differences were found among experimental groups
