# Supplementary material for: A Slow-Digesting Carbohydrate Diet during Rat Pregnancy Protects Offspring from Non-Alcoholic Fatty Liver Disease Risk through the Modulation of the Carbohydrate-Response Element and Sterol Regulatory Element Binding Proteins
Source: Nutrients. 2019 Apr 14;11(4):844. doi: 10.3390/nu11040844 (PMC6521114; doi:10.3390/nu11040844)
Supplement: Supplementary file 1 [file nutrients-11-00844-s001.zip › nutrients-454319-supplememtary/Supplemental Tables.pdf]

Supplemental Tables

Table S1. Experimental diets during rat pregnancy

	HF/SD Diet	HF/RD Diet	Reference
Total Protein (g/ 100 g diet)	24.2	24.2	18.3
Total Fat (g/ 100 g diet)	20.5	20.5	7.0
Total Carbohydrates (g/ 100 g diet)	49.1	49.4	64.6
Slow Digesting simple Sugars			
Isomaltulose[a]	67.9	-	-
Rapid Digesting simple Sugars			
Sucrose[a]	5.5	81.9	14.4
Rapid Digesting Complex Carbohydrates			
Maltodextrin (DE 9-16) [a]	7.7	-	78.4
Resistant Starch			
Resistant Maltodextrin Fibesol-2[a]	15.4	-	-
Indisgestible Fiber			
Fructooligosaccharides[a]	3.5	18.1	-
Cellulose[a]	-	-	7.2

[a] Individual carbohydrate composition is expressed as a percentage of total carbohydrates

Table S2. Glucose, insulin and glucagon levels in fasted serum samples from the different experimental groups (n=8):

	HF/SD Group	HF/RD Group	Reference
Glycaemia (mg/dL)	104.6 ± 5.0	119.0 ± 6.4	117.3 ± 12.8
Insulin (pg/mL)	885.2 ± 174.6	1319.0 ± 307.7	1234.2 ± 315.5
Glucagon (pg/mL)	29.4 ± 1.3	29.0 ± 2.1	22.5 ± 1.2

There are no significant differences among groups.

Table S3. Heat map showing relevant changes in liver lipids.

Individual Notation or Probable ID	Class	Subclass A	Mean HF/RD	SEM	Mean HF/SD	SEM	Student´s t-test (p)	Fold Change	Log2 (fold change)
12:0	Fatty acids	SFA	0.34	0.04	0.37	0.05	**6.51E-01**	0.92	-0.12
14:0	Fatty acids	SFA	0.37	0.02	0.39	0.03	**6.35E-01**	0.96	-0.06
16:0	Fatty acids	SFA	0.80	0.05	0.82	0.04	**7.90E-01**	0.98	-0.03
17:0	Fatty acids	SFA	0.63	0.05	0.79	0.05	**2.95E-02**	0.79	-0.34
18:0	Fatty acids	SFA	0.96	0.04	0.92	0.05	**5.06E-01**	1.05	0.07
16:1n-x	Fatty acids	MUFA	0.80	0.19	0.54	0.05	**2.38E-01**	1.47	0.56
16:3n-x	Fatty acids	MUFA	0.64	0.09	0.61	0.06	**8.21E-01**	1.04	0.06
18:2n-x	Fatty acids	MUFA	0.97	0.07	1.09	0.17	**5.47E-01**	0.90	-0.16
20:2n-x	Fatty acids	MUFA	0.23	0.04	0.26	0.04	**5.34E-01**	0.86	-0.22
20:3n-x	Fatty acids	MUFA	0.13	0.01	0.20	0.02	7.81E-03	0.65	-0.62
20:4n-x	Fatty acids	PUFA	0.19	0.08	0.24	0.03	**5.61E-01**	0.80	-0.32
AC(8:1n-x)	Fatty esters	PUFA	0.34	0.02	0.58	0.05	**7.12E-04**	0.57	-0.80
AC(10:1n-x)	Fatty esters	PUFA	0.77	0.08	0.63	0.07	**2.08E-01**	1.22	0.28
PE(18:3/0:0)	Glycerophospholipids	PUFA	0.80	0.24	1.32	0.31	**2.10E-01**	0.60	-0.73
PE(18:3/0:0)	Glycerophospholipids	PUFA	1.45	0.27	2.08	0.24	**1.08E-01**	0.70	-0.52
LPE(20:5)	Glycerophospholipids	PUFA	1.01	0.08	1.27	0.07	**3.95E-02**	0.80	-0.32
PE(22:5/0:0)	Glycerophospholipids	PUFA	0.92	0.05	0.90	0.05	**7.47E-01**	1.03	0.04
PE(22:5/0:0)	Glycerophospholipids	PUFA	1.32	0.26	1.49	0.16	**5.94E-01**	0.89	-0.17
PE(22:5/0:0)	Glycerophospholipids	PUFA	0.63	0.09	0.51	0.06	**2.86E-01**	1.24	0.31
PE(22:5/0:0)	Glycerophospholipids	PUFA	0.92	0.24	0.78	0.16	**6.23E-01**	1.19	0.25
PE(0:0/16:1)	Glycerophospholipids	PUFA	1.81	0.16	1.91	0.19	**6.97E-01**	0.95	-0.08
11-HETE	Oxidized fatty acids	PUFA	0.43	0.03	0.43	0.04	**9.97E-01**	1.00	0.00
12-HETE	Oxidized fatty acids	PUFA	0.03	0.01	0.03	0.01	**5.38E-01**	0.80	-0.32
13-HODE	Oxidized fatty acids	PUFA	0.20	0.01	0.28	0.02	1.71E-03	0.71	-0.50
15-HETE	Oxidized fatty acids	PUFA	0.16	0.02	0.16	0.01	**7.58E-01**	1.04	0.06
16:1n-7	Fatty acids	PUFA	1.73	0.24	1.56	0.12	**5.50E-01**	1.11	0.15
16:1n-9	Fatty acids	PUFA	0.78	0.08	1.14	0.11	**2.47E-02**	0.69	-0.54
18:1n-9	Fatty acids	PUFA	0.76	0.07	0.93	0.06	**8.10E-02**	0.81	-0.30
18:2n-6	Fatty acids	oxFA	0.51	0.05	0.85	0.07	1.64E-03	0.60	-0.74
18:3n-3	Fatty acids	oxFA	0.52	0.08	0.82	0.06	8.25E-03	0.64	-0.65
18:3n-6	Fatty acids	oxFA	0.66	0.08	0.93	0.07	**2.50E-02**	0.71	-0.49
18:4n-3	Fatty acids	oxFA	0.66	0.10	1.39	0.19	5.04E-03	0.47	-1.07
20:1n-6	Fatty acids	oxFA	0.47	0.01	0.65	0.05	8.55E-03	0.72	-0.47
20:3n-3	Fatty acids	oxFA	0.37	0.02	0.34	0.02	**4.47E-01**	1.06	0.09
20:3n-9	Fatty acids	oxFA	0.38	0.03	0.41	0.05	**5.21E-01**	0.91	-0.14
20:4n-3	Fatty acids	AC	0.10	0.01	0.17	0.02	4.85E-03	0.57	-0.80

20:4n-6	Fatty acids	AC	0.64	0.02	0.75	0.05	**8.31E-02**	0.85	-0.23
20:5n-3	Fatty acids	DG	0.55	0.07	0.76	0.04	**1.67E-02**	0.73	-0.46
22:4n-6	Fatty acids	DG	0.25	0.03	0.20	0.02	**1.60E-01**	1.25	0.32
22:5n-3	Fatty acids	DG	0.30	0.01	0.34	0.02	**2.02E-01**	0.90	-0.16
22:5n-6	Fatty acids	DG	0.27	0.04	0.23	0.03	**5.14E-01**	1.14	0.18
22:6n-3	Fatty acids	DG	0.63	0.04	0.64	0.04	**8.33E-01**	0.98	-0.03
Cer(42:3)	Sphingolipids	DG	0.49	0.03	0.53	0.03	**3.12E-01**	0.92	-0.12
Cer(d18:1/16:0)	Sphingolipids	DG	0.97	0.06	0.93	0.06	**7.01E-01**	1.04	0.05
Cer(d18:1/23:0)	Sphingolipids	DG	0.96	0.06	1.00	0.08	**7.08E-01**	0.96	-0.05
Cer(d18:1/24:0)	Sphingolipids	DG	0.93	0.05	0.93	0.07	**9.44E-01**	0.99	-0.01
Cer(d18:1/24:1)	Sphingolipids	DG	1.08	0.06	0.99	0.08	**3.98E-01**	1.09	0.12
CMH(d18:1/22:0)	Sphingolipids	TG	0.68	0.05	0.62	0.04	**3.29E-01**	1.10	0.14
CMH(d18:1/24:0)	Sphingolipids	TG	0.70	0.08	0.80	0.06	**3.25E-01**	0.88	-0.19
ChoE(20:4)	Sterols	TG	1.31	0.08	1.17	0.15	**4.06E-01**	1.12	0.16
ChoE(20:5)	Sterols	TG	1.45	0.11	1.63	0.29	**5.95E-01**	0.90	-0.16
ChoE(22:4)	Sterols	TG	0.87	0.09	1.15	0.23	**2.80E-01**	0.75	-0.41
ChoE(22:5)	Sterols	TG	0.67	0.07	1.07	0.26	**1.83E-01**	0.62	-0.68
ChoE(22:6)	Sterols	TG	1.15	0.13	1.12	0.16	**8.52E-01**	1.04	0.05
Cholic acid	Bile acids	TG	2.06	1.34	2.94	2.69	**7.75E-01**	0.70	-0.51
DG(32:0)	Glycerolipids	TG	1.89	0.31	0.73	0.08	**6.87E-03**	2.60	1.38
DG(32:1)	Glycerolipids	TG	1.68	0.28	0.78	0.10	**1.61E-02**	2.15	1.10
DG(32:2)	Glycerolipids	TG	0.78	0.11	0.55	0.07	**9.28E-02**	1.44	0.52
DG(34:0)	Glycerolipids	TG	1.13	0.08	0.80	0.04	**4.71E-03**	1.42	0.50
DG(34:1)	Glycerolipids	TG	1.69	0.23	0.97	0.09	**1.77E-02**	1.75	0.81
DG(34:2)	Glycerolipids	TG	1.12	0.12	1.01	0.05	**4.33E-01**	1.11	0.15
DG(36:2)	Glycerolipids	TG	0.97	0.07	1.00	0.08	**7.86E-01**	0.97	-0.04
DG(36:3)	Glycerolipids	TG	0.64	0.07	1.02	0.06	**1.26E-03**	0.63	-0.68
DG(36:4)	Glycerolipids	TG	0.44	0.09	0.87	0.11	**7.19E-03**	0.50	-0.99
DG(36:4)	Glycerolipids	TG	1.17	0.08	1.19	0.10	**9.28E-01**	0.99	-0.01
Hydroxy-eicosatetraenoic acids (HETE)	Oxidized fatty acids	TG	0.62	0.05	0.62	0.04	**9.95E-01**	1.00	0.00
PC(0:0/14:0)	Glycerophospholipids	TG	0.33	0.04	0.43	0.04	**9.70E-02**	0.76	-0.39
PC(0:0/15:0)	Glycerophospholipids	TG	0.78	0.07	1.18	0.05	**5.34E-04**	0.66	-0.59
PC(0:0/16:1)	Glycerophospholipids	TG	1.74	0.17	1.59	0.18	**5.47E-01**	1.09	0.13
PC(0:0/18:0)	Glycerophospholipids	TG	1.26	0.14	1.35	0.10	**6.52E-01**	0.94	-0.09
PC(0:0/18:1)	Glycerophospholipids	TG	1.26	0.06	1.26	0.08	**9.99E-01**	1.00	0.00
PC(0:0/18:2)	Glycerophospholipids	TG	0.77	0.06	1.06	0.03	**1.08E-03**	0.72	-0.48
PC(0:0/18:3)	Glycerophospholipids	TG	0.60	0.05	0.75	0.08	**1.27E-01**	0.80	-0.32
PC(0:0/20:0)	Glycerophospholipids	TG	0.75	0.11	1.07	0.10	**4.75E-02**	0.70	-0.51
PC(0:0/20:2)	Glycerophospholipids	TG	0.57	0.03	0.67	0.04	**1.12E-01**	0.86	-0.21
PC(0:0/20:3)	Glycerophospholipids	TG	0.94	0.09	0.96	0.09	**8.49E-01**	0.97	-0.04

PC(0:0/20:4)	Glycerophospholipids	TG	1.14	0.06	1.12	0.05	**8.92E-01**	1.01	0.01
PC(0:0/20:5)	Glycerophospholipids	TG	1.13	0.11	1.17	0.08	**7.70E-01**	0.96	-0.05
PC(0:0/22:4)	Glycerophospholipids	TG	0.79	0.08	0.61	0.04	**5.31E-02**	1.31	0.39
PC(0:0/22:5)	Glycerophospholipids	TG	1.16	0.20	1.21	0.18	**8.57E-01**	0.96	-0.06
PC(0:0/22:5)	Glycerophospholipids	TG	0.77	0.14	0.45	0.04	**6.75E-02**	1.71	0.77
PC(0:0/22:6)	Glycerophospholipids	TG	0.90	0.13	0.69	0.04	**1.68E-01**	1.31	0.39
PC(14:0/0:0)	Glycerophospholipids	TG	0.34	0.03	0.41	0.04	**2.10E-01**	0.84	-0.25
PC(14:0/18:2)	Glycerophospholipids	TG	0.25	0.02	0.35	0.04	**6.02E-02**	0.73	-0.45
PC(14:0/20:4)	Glycerophospholipids	TG	0.41	0.04	0.49	0.04	**1.64E-01**	0.83	-0.27
PC(15:0/0:0)	Glycerophospholipids	TG	0.74	0.06	1.03	0.04	**1.09E-03**	0.72	-0.47
PC(15:0/18:2)	Glycerophospholipids	TG	0.67	0.12	1.19	0.10	**5.54E-03**	0.56	-0.84
PC(15:0/20:4)	Glycerophospholipids	TG	0.90	0.08	1.34	0.09	**2.66E-03**	0.67	-0.57
PC(15:0/22:6)	Glycerophospholipids	TG	0.45	0.04	0.61	0.05	**2.84E-02**	0.74	-0.43
PC(16:0/0:0)	Glycerophospholipids	TG	1.00	0.04	1.07	0.04	**2.53E-01**	0.94	-0.09
PC(16:0/16:0)	Glycerophospholipids	TG	0.82	0.03	0.88	0.04	**2.29E-01**	0.93	-0.11
PC(16:0/17:0)	Glycerophospholipids	TG	1.19	0.07	1.44	0.16	**1.72E-01**	0.83	-0.27
PC(16:0/18:0)	Glycerophospholipids	TG	0.93	0.05	0.95	0.03	**8.05E-01**	0.98	-0.02
PC(16:0/18:1)	Glycerophospholipids	TG	1.21	0.08	1.17	0.07	**6.98E-01**	1.04	0.05
PC(16:0/18:2)	Glycerophospholipids	TG	0.90	0.07	1.11	0.04	**2.98E-02**	0.81	-0.30
PC(16:0/19:1)	Glycerophospholipids	TG	0.74	0.04	0.94	0.05	**1.04E-02**	0.79	-0.35
PC(16:0/20:4)	Glycerophospholipids	TG	1.11	0.07	1.10	0.02	**8.85E-01**	1.01	0.01
PC(16:0/20:5)	Glycerophospholipids	TG	1.03	0.13	1.41	0.07	**2.88E-02**	0.73	-0.45
PC(16:0/22:6)	Glycerophospholipids	TG	0.78	0.07	0.72	0.04	**4.81E-01**	1.08	0.11
PC(16:1/0:0)	Glycerophospholipids	TG	1.65	0.16	1.68	0.19	**9.16E-01**	0.98	-0.02
PC(17:0/18:2)	Glycerophospholipids	TG	0.55	0.10	1.05	0.10	**3.77E-03**	0.52	-0.94
PC(17:0/20:3)	Glycerophospholipids	TG	0.58	0.06	0.88	0.11	**3.36E-02**	0.66	-0.60
PC(17:0/20:4)	Glycerophospholipids	TG	1.21	0.12	1.81	0.18	**1.39E-02**	0.67	-0.58
PC(17:0/20:4)	Glycerophospholipids	TG	0.89	0.08	1.23	0.10	**1.99E-02**	0.72	-0.47
PC(17:1/18:1)	Glycerophospholipids	TG	0.83	0.12	1.68	0.27	**9.84E-03**	0.49	-1.02
PC(17:1/18:2)	Glycerophospholipids	TG	0.67	0.14	1.21	0.13	**1.43E-02**	0.55	-0.86
PC(18:0/0:0)	Glycerophospholipids	TG	1.07	0.04	1.05	0.05	**7.89E-01**	1.02	0.02
PC(18:0/18:1)	Glycerophospholipids	TG	1.31	0.08	1.16	0.09	**2.82E-01**	1.12	0.17
PC(18:0/18:2)	Glycerophospholipids	TG	0.74	0.05	0.96	0.05	**1.04E-02**	0.77	-0.37
PC(18:0/20:3)	Glycerophospholipids	TG	0.79	0.07	0.86	0.08	**5.43E-01**	0.92	-0.12
PC(18:0/20:4)	Glycerophospholipids	TG	1.09	0.04	1.03	0.03	**2.73E-01**	1.06	0.09
PC(18:0/22:4)	Glycerophospholipids	TG	0.53	0.05	0.47	0.03	**3.23E-01**	1.13	0.18
PC(18:0/22:5)	Glycerophospholipids	TG	0.70	0.04	0.78	0.03	**1.32E-01**	0.90	-0.15
PC(18:0/22:6)	Glycerophospholipids	TG	0.95	0.09	0.75	0.02	**7.16E-02**	1.27	0.34
PC(18:1/0:0)	Glycerophospholipids	TG	1.12	0.06	1.40	0.06	**5.52E-03**	0.80	-0.33
PC(18:1/22:6)	Glycerophospholipids	ChoE	1.10	0.07	1.21	0.06	**2.79E-01**	0.91	-0.13
PC(18:2/0:0)	Glycerophospholipids	ChoE	0.76	0.13	1.00	0.05	**1.25E-01**	0.76	-0.39

PC(18:2/0:0)	Glycerophospholipids	ChoE	1.00	0.13	1.01	0.08	**9.74E-01**	0.99	-0.01
PC(18:2/18:2)	Glycerophospholipids	ChoE	0.43	0.11	0.82	0.07	**1.14E-02**	0.52	-0.94
PC(18:2/20:4)	Glycerophospholipids	ChoE	0.63	0.09	0.80	0.08	**1.81E-01**	0.79	-0.34
PC(18:3/0:0)	Glycerophospholipids	BA	1.18	0.26	1.26	0.11	**7.85E-01**	0.94	-0.09
PC(18:3/18:3)	Glycerophospholipids	BA	0.22	0.03	0.23	0.02	**7.89E-01**	0.96	-0.07
PC(19:1/18:2)	Glycerophospholipids	BA	0.54	0.12	1.24	0.17	**4.33E-03**	0.44	-1.20
PC(20:0/0:0)	Glycerophospholipids	PE	0.66	0.02	0.90	0.05	**7.15E-04**	0.74	-0.44
PC(20:0/18:2)	Glycerophospholipids	PE	0.52	0.02	0.70	0.05	**3.54E-03**	0.74	-0.44
PC(20:0/20:4)	Glycerophospholipids	PE	1.07	0.06	1.48	0.14	**1.53E-02**	0.72	-0.46
PC(20:1/0:0)	Glycerophospholipids	PE	0.86	0.04	1.21	0.07	**1.28E-03**	0.71	-0.49
PC(20:2/0:0)	Glycerophospholipids	PE	0.41	0.03	0.53	0.02	**7.90E-03**	0.77	-0.38
PC(20:3/0:0)	Glycerophospholipids	PE	0.93	0.19	1.01	0.10	**7.01E-01**	0.92	-0.12
PC(20:3/0:0)	Glycerophospholipids	PE	0.85	0.11	0.94	0.11	**5.74E-01**	0.91	-0.14
PC(20:4/0:0)	Glycerophospholipids	PE	1.54	0.31	1.56	0.16	**9.52E-01**	0.99	-0.02
PC(20:5/0:0)	Glycerophospholipids	PE	1.34	0.39	1.49	0.16	**7.23E-01**	0.90	-0.16
PC(22:4/0:0)	Glycerophospholipids	PE	0.74	0.16	0.72	0.11	**8.89E-01**	1.04	0.05
PC(22:4/20:4)	Glycerophospholipids	PE	0.31	0.03	0.34	0.02	**3.32E-01**	0.89	-0.17
PC(22:5/0:0)	Glycerophospholipids	PE	0.89	0.20	0.93	0.13	**8.90E-01**	0.96	-0.05
PC(22:5/0:0)	Glycerophospholipids	PE	0.77	0.22	0.66	0.14	**6.77E-01**	1.17	0.22
PC(22:6/0:0)	Glycerophospholipids	PE	1.16	0.28	1.09	0.15	**8.32E-01**	1.06	0.09
PC(32:1)	Glycerophospholipids	PE	1.39	0.11	1.14	0.14	**1.84E-01**	1.22	0.28
PC(33:1)	Glycerophospholipids	PE	1.07	0.08	1.32	0.10	**6.67E-02**	0.81	-0.30
PC(37:2)	Glycerophospholipids	PE	0.95	0.16	2.14	0.47	**4.42E-02**	0.44	-1.18
PC(37:2)	Glycerophospholipids	PE	0.50	0.08	1.01	0.09	**1.05E-03**	0.50	-1.00
PC(37:5)	Glycerophospholipids	PE	0.76	0.11	1.13	0.08	**1.73E-02**	0.67	-0.57
PC(38:5)	Glycerophospholipids	PE	0.94	0.05	1.13	0.06	**2.25E-02**	0.83	-0.26
PC(38:5)	Glycerophospholipids	LPE	0.43	0.07	0.35	0.07	**4.83E-01**	1.20	0.26
PC(40:5)	Glycerophospholipids	LPE	0.60	0.09	0.39	0.04	**6.95E-02**	1.53	0.62
PC(40:8)	Glycerophospholipids	LPE	0.95	0.06	0.98	0.15	**8.65E-01**	0.97	-0.04
PC(O-16:0/0:0)	Glycerophospholipids	LPE	0.87	0.06	0.90	0.07	**8.11E-01**	0.98	-0.04
PC(O-16:0/14:0)	Glycerophospholipids	LPE	0.22	0.03	0.23	0.02	**5.91E-01**	0.92	-0.12
PC(O-16:0/16:0)	Glycerophospholipids	LPE	0.97	0.04	1.04	0.03	**2.04E-01**	0.93	-0.10
PC(O-16:0/18:2)	Glycerophospholipids	LPE	0.53	0.04	0.68	0.05	**3.31E-02**	0.78	-0.36
PC(O-16:0/20:4)	Glycerophospholipids	LPE	0.85	0.05	0.86	0.03	**8.56E-01**	0.99	-0.02
PC(O-16:0/22:4)	Glycerophospholipids	LPE	0.73	0.06	0.59	0.04	**7.37E-02**	1.23	0.30
PC(O-18:0/0:0)	Glycerophospholipids	LPE	0.88	0.05	0.90	0.07	**8.01E-01**	0.98	-0.04
PC(O-18:0/20:4)	Glycerophospholipids	LPE	0.91	0.05	0.91	0.03	**9.51E-01**	1.00	0.01
PC(O-18:1/0:0)	Glycerophospholipids	LPE	0.82	0.05	0.86	0.07	**6.59E-01**	0.95	-0.07
PC(O-18:1/18:2)	Glycerophospholipids	LPE	0.50	0.04	0.64	0.04	**2.00E-02**	0.77	-0.38
PC(O-18:1/20:4)	Glycerophospholipids	LPE	0.83	0.04	0.88	0.02	**3.96E-01**	0.95	-0.07
PC(O-18:1/20:4)	Glycerophospholipids	LPE	0.85	0.07	0.89	0.03	**6.33E-01**	0.96	-0.06

PC(O-18:1/22:4)	Glycerophospholipids	LPE	0.60	0.06	0.55	0.05	**5.18E-01**	1.09	0.13
PC(O-20:0/0:0)	Glycerophospholipids	LPE	0.90	0.07	0.83	0.04	**4.30E-01**	1.08	0.11
PC(O-34:0)	Glycerophospholipids	LPE	1.04	0.05	0.99	0.06	**5.30E-01**	1.05	0.07
PC(O-34:1)	Glycerophospholipids	LPE	0.83	0.03	0.89	0.03	**1.42E-01**	0.93	-0.10
PC(P-16:0/0:0)	Glycerophospholipids	LPE	0.79	0.08	0.86	0.06	**4.53E-01**	0.91	-0.13
PC(P-16:0/16:0)	Glycerophospholipids	LPE	0.91	0.05	1.03	0.04	**7.69E-02**	0.89	-0.17
PC(P-16:0/20:4)	Glycerophospholipids	LPE	0.73	0.07	0.86	0.03	**9.39E-02**	0.84	-0.25
PC(P-16:0/22:6)	Glycerophospholipids	LPE	0.44	0.08	0.40	0.03	**7.15E-01**	1.08	0.11
PC(P-18:0/20:4)	Glycerophospholipids	LPE	0.57	0.06	0.57	0.06	**9.81E-01**	1.00	0.01
PC(P-18:0/20:4)	Glycerophospholipids	LPE	0.89	0.09	0.85	0.06	**7.20E-01**	1.05	0.07
PE(0:0/16:0)	Glycerophospholipids	LPE	1.37	0.23	1.74	0.22	**2.63E-01**	0.79	-0.35
PE(0:0/18:0)	Glycerophospholipids	LPE	1.32	0.23	1.56	0.23	**4.77E-01**	0.85	-0.24
PE(0:0/18:2)	Glycerophospholipids	LPE	0.88	0.11	1.51	0.09	9.97E-04	0.58	-0.77
PE(0:0/20:4)	Glycerophospholipids	PC	1.07	0.04	1.17	0.04	**1.26E-01**	0.92	-0.13
PE(0:0/22:6)	Glycerophospholipids	PC	1.05	0.09	0.93	0.06	**3.10E-01**	1.13	0.17
PE(14:0/0:0)	Glycerophospholipids	PC	0.38	0.02	0.51	0.04	**1.75E-02**	0.76	-0.40
PE(16:0/0:0)	Glycerophospholipids	PC	1.10	0.08	1.23	0.06	**2.14E-01**	0.89	-0.16
PE(16:0/18:1)	Glycerophospholipids	PC	1.46	0.08	1.62	0.16	**3.66E-01**	0.90	-0.15
PE(16:0/18:2)	Glycerophospholipids	PC	1.07	0.13	1.68	0.13	**5.16E-03**	0.64	-0.65
PE(16:0/20:4)	Glycerophospholipids	PC	1.27	0.06	1.30	0.05	**7.31E-01**	0.98	-0.03
PE(16:0/22:6)	Glycerophospholipids	PC	0.92	0.07	0.82	0.05	**2.62E-01**	1.12	0.17
PE(18:0/0:0)	Glycerophospholipids	PC	1.13	0.10	1.14	0.06	**9.38E-01**	0.99	-0.01
PE(18:0/20:4)	Glycerophospholipids	PC	1.05	0.04	1.03	0.03	**7.65E-01**	1.02	0.02
PE(18:0/22:6)	Glycerophospholipids	PC	1.12	0.11	0.84	0.06	**5.64E-02**	1.33	0.41
PE(18:1/0:0)	Glycerophospholipids	PC	1.33	0.11	1.92	0.10	**2.54E-03**	0.70	-0.52
PE(18:1/18:2)	Glycerophospholipids	PC	0.81	0.13	1.75	0.22	**2.36E-03**	0.46	-1.11
PE(18:1e/22:4)	Glycerophospholipids	PC	0.79	0.03	0.74	0.04	**4.15E-01**	1.06	0.09
PE(18:2/0:0)	Glycerophospholipids	PC	1.09	0.25	1.99	0.20	**1.63E-02**	0.55	-0.87
PE(18:2/0:0)	Glycerophospholipids	PC	1.04	0.12	1.73	0.14	**3.05E-03**	0.60	-0.73
PE(18:2/18:2)	Glycerophospholipids	PC	0.51	0.16	1.07	0.09	**1.25E-02**	0.48	-1.06
PE(20:3/0:0)	Glycerophospholipids	PC	1.20	0.28	1.75	0.17	**1.22E-01**	0.69	-0.54
PE(20:4/0:0)	Glycerophospholipids	PC	1.44	0.24	1.71	0.15	**3.56E-01**	0.84	-0.25
PE(20:5/16:0)	Glycerophospholipids	PC	1.24	0.12	1.62	0.11	**3.48E-02**	0.76	-0.39
PE(22:4/0:0)	Glycerophospholipids	PC	0.88	0.17	1.00	0.14	**5.87E-01**	0.88	-0.18
PE(22:6/0:0)	Glycerophospholipids	PC	1.29	0.23	1.49	0.19	**5.13E-01**	0.86	-0.21
PE(38:5)	Glycerophospholipids	PC	1.14	0.06	1.33	0.07	**6.27E-02**	0.86	-0.22
PE(O-16:0/0:0)	Glycerophospholipids	PC	0.57	0.04	0.64	0.07	**4.16E-01**	0.89	-0.17
PE(O-16:0/18:1)	Glycerophospholipids	PC	0.86	0.05	1.04	0.06	**3.61E-02**	0.82	-0.28
PE(P-16:0/18:2)	Glycerophospholipids	PC	0.67	0.06	1.04	0.09	**3.15E-03**	0.64	-0.64
PE(P-16:0/20:4)	Glycerophospholipids	PC	0.76	0.03	0.83	0.04	**2.28E-01**	0.92	-0.12
PE(P-16:0/22:6)	Glycerophospholipids	PC	0.61	0.05	0.61	0.04	**9.74E-01**	1.00	0.00

PE(P-16:1/0:0)	Glycerophospholipids	PC	0.86	0.10	1.18	0.12	**6.31E-02**	0.73	-0.46
PE(P-18:0/0:0)	Glycerophospholipids	PC	1.04	0.11	1.08	0.06	**7.27E-01**	0.96	-0.06
PE(P-18:0/18:1)	Glycerophospholipids	PC	1.07	0.07	1.12	0.08	**6.48E-01**	0.95	-0.07
PE(P-18:0/20:4)	Glycerophospholipids	PC	1.04	0.04	1.02	0.04	**8.01E-01**	1.01	0.02
PE(P-18:0/22:5) + PE(P-20:1/20:4)	Glycerophospholipids	PC	0.98	0.04	0.97	0.05	**9.19E-01**	1.01	0.01
PE(P-18:1/0:0)	Glycerophospholipids	PC	0.88	0.09	1.21	0.10	**3.28E-02**	0.73	-0.46
PE(P-18:1/20:4)	Glycerophospholipids	PC	0.85	0.03	0.90	0.03	**2.25E-01**	0.94	-0.09
PE(P-18:2/0:0)	Glycerophospholipids	PC	0.40	0.06	0.71	0.09	9.27E-03	0.56	-0.84
PE(P-20:0/20:4)	Glycerophospholipids	PC	0.95	0.08	0.75	0.06	**7.37E-02**	1.26	0.33
PI(16:0/18:1)	Glycerophospholipids	PC	1.05	0.05	0.98	0.04	**3.44E-01**	1.07	0.09
PI(16:0/18:2)	Glycerophospholipids	PC	1.15	0.08	1.21	0.08	**6.49E-01**	0.95	-0.07
PI(16:0/20:4)	Glycerophospholipids	PC	0.97	0.04	1.03	0.05	**3.33E-01**	0.94	-0.09
PI(18:0/18:2)	Glycerophospholipids	PC	0.97	0.11	0.96	0.07	**9.32E-01**	1.01	0.02
PI(18:0/20:3)	Glycerophospholipids	PC	1.25	0.15	1.36	0.20	**6.64E-01**	0.92	-0.12
PI(18:0/20:4)	Glycerophospholipids	PC	1.06	0.05	1.02	0.05	**5.30E-01**	1.04	0.06
PI(18:0/22:6)	Glycerophospholipids	PC	1.38	0.14	0.99	0.13	**6.80E-02**	1.39	0.47
PI(18:1/20:4)	Glycerophospholipids	PC	0.75	0.10	0.76	0.06	**9.36E-01**	0.99	-0.02
SM(32:1)	Sphingolipids	PC	0.58	0.02	0.58	0.02	**9.59E-01**	1.00	0.00
SM(33:1)	Sphingolipids	PC	1.03	0.08	1.37	0.08	7.37E-03	0.75	-0.42
SM(38:1)	Sphingolipids	PC	0.99	0.06	1.07	0.04	**3.05E-01**	0.93	-0.11
SM(39:1)	Sphingolipids	PC	1.04	0.10	1.34	0.05	**2.51E-02**	0.78	-0.36
SM(42:1)	Sphingolipids	PC	0.99	0.08	1.17	0.06	**9.57E-02**	0.85	-0.24
SM(42:3)	Sphingolipids	PC	0.56	0.03	0.73	0.04	6.73E-03	0.77	-0.37
SM(d16:1/24:1)	Sphingolipids	PC	1.39	0.13	1.69	0.14	**1.57E-01**	0.83	-0.28
SM(d18:0/16:0)	Sphingolipids	PC	1.13	0.10	1.34	0.08	**1.22E-01**	0.84	-0.25
SM(d18:0/18:0)	Sphingolipids	PC	0.78	0.07	0.74	0.05	**6.23E-01**	1.06	0.08
SM(d18:0/22:0)	Sphingolipids	PC	0.97	0.07	1.25	0.09	**2.36E-02**	0.78	-0.37
SM(d18:1/16:0)	Sphingolipids	PC	1.14	0.05	1.20	0.03	**3.09E-01**	0.95	-0.07
SM(d18:1/17:0)	Sphingolipids	PC	0.71	0.08	0.97	0.14	**1.12E-01**	0.74	-0.44
SM(d18:1/18:0)	Sphingolipids	PC	0.78	0.05	0.75	0.03	**5.76E-01**	1.04	0.06
SM(d18:1/22:0)	Sphingolipids	PC	1.13	0.06	1.21	0.04	**2.56E-01**	0.93	-0.11
SM(d18:1/23:0)	Sphingolipids	PC	1.04	0.07	1.29	0.04	**1.03E-02**	0.81	-0.31
SM(d18:1/23:1)	Sphingolipids	PC	1.32	0.13	1.71	0.11	**5.04E-02**	0.78	-0.37
SM(d18:1/24:1)	Sphingolipids	LPC	1.03	0.06	1.17	0.04	**8.44E-02**	0.88	-0.18
SM(d18:2/16:0)	Sphingolipids	LPC	0.71	0.03	0.79	0.03	**8.63E-02**	0.90	-0.15
SM(d18:2/20:0)	Sphingolipids	LPC	0.70	0.06	0.90	0.06	**3.40E-02**	0.78	-0.35
SM(d18:2/22:0)	Sphingolipids	LPC	0.39	0.04	0.41	0.05	**8.38E-01**	0.97	-0.05
SM(d18:2/23:0)	Sphingolipids	LPC	0.55	0.03	0.75	0.05	4.90E-03	0.73	-0.46
Taurolithocholic acid	Bile acids	LPC	1.70	0.34	1.43	0.25	**5.31E-01**	1.19	0.25
Tauroursodeoxycholic acid	Bile acids	LPC	1.94	0.17	1.52	0.18	**1.11E-01**	1.28	0.35

TG(42:0)	Glycerolipids	LPC	0.08	0.01	0.05	0.01	5.25E-03	1.56	0.64
TG(42:1)	Glycerolipids	LPC	0.11	0.01	0.06	0.00	9.01E-03	1.88	0.91
TG(44:0)	Glycerolipids	LPC	0.34	0.03	0.16	0.02	4.79E-04	2.04	1.03
TG(44:1)	Glycerolipids	LPC	0.23	0.04	0.10	0.01	7.55E-03	2.32	1.21
TG(44:2)	Glycerolipids	LPC	0.09	0.01	0.05	0.00	1.21E-02	1.73	0.79
TG(45:0)	Glycerolipids	LPC	1.07	0.06	0.87	0.03	9.21E-03	1.24	0.31
TG(46:0)	Glycerolipids	LPC	1.36	0.20	0.43	0.06	1.82E-03	3.15	1.66
TG(46:1)	Glycerolipids	LPC	1.14	0.21	0.34	0.04	6.31E-03	3.36	1.75
TG(46:2)	Glycerolipids	LPC	0.48	0.11	0.19	0.01	3.56E-02	2.51	1.33
TG(46:3)	Glycerolipids	LPC	0.26	0.04	0.19	0.02	2.07E-01	1.35	0.44
TG(47:0)	Glycerolipids	LPC	1.67	0.30	0.90	0.07	4.08E-02	1.84	0.88
TG(47:1)	Glycerolipids	LPC	1.95	0.43	0.90	0.07	4.39E-02	2.17	1.12
TG(48:0)	Glycerolipids	LPC	2.76	0.40	0.61	0.13	7.74E-04	4.53	2.18
TG(48:1)	Glycerolipids	LPC	2.54	0.38	0.65	0.13	1.13E-03	3.91	1.97
TG(48:2)	Glycerolipids	LPC	2.20	0.42	0.69	0.11	8.16E-03	3.19	1.67
TG(48:3)	Glycerolipids	LPC	0.95	0.22	0.55	0.04	1.17E-01	1.73	0.79
TG(48:4)	Glycerolipids	LPC	0.19	0.04	0.23	0.03	4.90E-01	0.84	-0.25
TG(49:0)	Glycerolipids	LPC	2.53	0.54	0.88	0.11	1.81E-02	2.88	1.53
TG(49:1)	Glycerolipids	LPC	2.81	0.71	1.07	0.12	4.43E-02	2.62	1.39
TG(49:2)	Glycerolipids	LPC	2.33	0.58	1.24	0.10	1.06E-01	1.87	0.91
TG(49:3)	Glycerolipids	LPC	1.73	0.41	1.45	0.07	5.29E-01	1.19	0.25
TG(50:0)	Glycerolipids	LPC	2.99	0.64	0.57	0.09	6.57E-03	5.22	2.38
TG(50:1)	Glycerolipids	LPC	2.26	0.26	0.91	0.12	5.99E-04	2.48	1.31
TG(50:2)	Glycerolipids	LPC	2.12	0.23	1.08	0.13	2.59E-03	1.96	0.97
TG(50:3)	Glycerolipids	LPC	1.81	0.24	1.24	0.10	5.77E-02	1.46	0.54
TG(50:4)	Glycerolipids	LPC	1.09	0.25	0.96	0.06	6.22E-01	1.14	0.19
TG(50:5)	Glycerolipids	LPC	0.63	0.12	0.69	0.05	6.38E-01	0.91	-0.14
TG(51:1)	Glycerolipids	LPC	2.65	0.57	1.15	0.14	3.40E-02	2.30	1.20
TG(51:2)	Glycerolipids	LPC	2.57	0.66	1.45	0.16	1.41E-01	1.77	0.82
TG(51:3)	Glycerolipids	LPC	1.68	0.43	1.74	0.19	9.00E-01	0.97	-0.05
TG(51:4)	Glycerolipids	LPC	1.18	0.25	1.74	0.21	1.16E-01	0.68	-0.56
TG(52:0)	Glycerolipids	LPC	1.41	0.30	0.61	0.05	3.22E-02	2.30	1.20
TG(52:1)	Glycerolipids	LPC	2.88	0.54	0.79	0.09	5.89E-03	3.63	1.86
TG(52:5)	Glycerolipids	LPC	1.31	0.27	1.74	0.15	2.10E-01	0.75	-0.41
TG(53:1)	Glycerolipids	PI	1.55	0.21	0.77	0.12	8.40E-03	2.02	1.02
TG(53:2)	Glycerolipids	PI	1.71	0.30	1.39	0.12	3.54E-01	1.22	0.29
TG(53:3)	Glycerolipids	PI	1.28	0.26	1.45	0.14	5.74E-01	0.88	-0.19
TG(53:4)	Glycerolipids	PI	0.99	0.18	1.51	0.16	5.76E-02	0.66	-0.60
TG(54:0)	Glycerolipids	PI	0.83	0.05	1.03	0.18	3.27E-01	0.81	-0.31
TG(54:1)	Glycerolipids	PI	1.19	0.18	0.71	0.06	3.45E-02	1.67	0.74
TG(54:2)	Glycerolipids	PI	1.70	0.32	1.01	0.10	7.36E-02	1.68	0.75

TG(54:3)	Glycerolipids	PI	1.08	0.10	1.23	0.09	**2.81E-01**	0.88	-0.19
TG(54:4)	Glycerolipids	Cer	0.77	0.08	1.24	0.12	**5.06E-03**	0.62	-0.69
TG(54:5)	Glycerolipids	Cer	0.69	0.09	1.39	0.16	**1.58E-03**	0.50	-1.00
TG(54:5)	Glycerolipids	Cer	0.95	0.22	0.75	0.09	**4.29E-01**	1.26	0.34
TG(54:6)	Glycerolipids	Cer	0.63	0.15	1.66	0.31	**8.35E-03**	0.38	-1.40
TG(54:6)	Glycerolipids	Cer	0.65	0.15	0.77	0.13	**5.46E-01**	0.84	-0.26
TG(54:7)	Glycerolipids	SM	0.71	0.18	1.95	0.40	**1.18E-02**	0.36	-1.47
TG(54:7)	Glycerolipids	SM	0.78	0.14	1.01	0.14	**2.70E-01**	0.77	-0.37
TG(56:0)	Glycerolipids	SM	0.93	0.05	0.97	0.05	**5.89E-01**	0.96	-0.06
TG(56:1)	Glycerolipids	SM	0.82	0.07	0.90	0.05	**4.18E-01**	0.92	-0.12
TG(56:2)	Glycerolipids	SM	0.76	0.13	0.83	0.08	**6.89E-01**	0.92	-0.12
TG(56:3)	Glycerolipids	SM	0.67	0.16	0.94	0.14	**2.41E-01**	0.72	-0.47
TG(56:5)	Glycerolipids	SM	0.63	0.11	0.88	0.11	**1.24E-01**	0.72	-0.48
TG(56:6)	Glycerolipids	SM	0.39	0.11	0.40	0.08	**9.56E-01**	0.98	-0.03
TG(56:7)	Glycerolipids	SM	0.34	0.08	0.62	0.12	**7.89E-02**	0.56	-0.83
TG(56:7)	Glycerolipids	SM	0.68	0.12	0.59	0.06	**5.08E-01**	1.17	0.22
TG(56:8)	Glycerolipids	SM	0.22	0.06	0.64	0.17	**4.95E-02**	0.35	-1.52
TG(56:8)	Glycerolipids	SM	0.62	0.14	0.74	0.12	**5.41E-01**	0.84	-0.25
TG(58:1)	Glycerolipids	SM	1.12	0.14	0.95	0.07	**3.26E-01**	1.18	0.23
TG(58:10)	Glycerolipids	SM	0.19	0.04	0.41	0.10	**6.61E-02**	0.47	-1.10
TG(58:2)	Glycerolipids	SM	0.91	0.13	0.95	0.12	**8.29E-01**	0.96	-0.06
TG(58:3)	Glycerolipids	SM	0.68	0.13	1.04	0.16	**1.06E-01**	0.66	-0.60
TG(58:6)	Glycerolipids	SM	0.22	0.06	0.23	0.04	**9.03E-01**	0.96	-0.06
TG(58:7)	Glycerolipids	SM	0.19	0.06	0.28	0.05	**2.82E-01**	0.68	-0.56
TG(58:9)	Glycerolipids	SM	0.23	0.05	0.41	0.09	**9.52E-02**	0.57	-0.80
TG(60:2)	Glycerolipids	SM	1.16	0.24	1.14	0.19	**9.50E-01**	1.02	0.03
TG(60:3)	Glycerolipids	SM	0.74	0.14	1.03	0.13	**1.56E-01**	0.72	-0.47
x-HETE/EET	Oxidized fatty acids	CMH	0.05	0.00	0.05	0.01	**7.78E-01**	0.94	-0.09
x-HODE	Oxidized fatty acids	CMH	0.38	0.06	0.54	0.05	**5.61E-02**	0.70	-0.51
x-HODE	Oxidized fatty acids	Hydroxy-octadecadienoic acids	0.76	0.14	1.15	0.17	**9.81E-02**	0.66	-0.60

Each metabolite is shown as a line whose color is defined by the sign and magnitude of the change. Adjacent column to each comparison shows the results of the t test (P value). Color scales are shown in the right: upper scale: Log_2 fold-change; lower scale: statistical significance (n: HF/SD=7, n: HF/RD=8). Cer, ceramides; ChoE, cholesterol esters; DAG, diglycerides; oxFFA, oxidized free fatty acids; LPC, lysophosphatidylcholines; LPE, lysophosphatidylethanolamines; PC, phosphatidylcholines; PE, phosphatidylethanolamines; PI, phosphatidylinositols; MUFA, monounsaturated fatty acids; PUFA, polyunsaturated fatty acids; SFA, saturated fatty acids; SM, sphingomyelins; TAG, triacylglycerols.

"Individual notation" refers to the confirmed identification of the metabolites. Overlapping of two or more metabolites or non-confirmed identification is indicated in "Individual composition (or probable ID)". Fold-changes and unpaired Student's t test *p*-values (or Welch´s t test where unequal variances were found) were calculated for each comparison considering 3 cell cultures per group. Regarding sphingolipids, dA:B represents the sphingoid base: d18:1, sphingosine; d18:2, sphingadiene; d18:0, sphinganine. For glycerophospholipids containing an ether moiety the prefix, O-, denotes the presence of an alkyl ether (plasmanyl) substituent, whereas the prefix P- refers to a vinyl ether (alkenyl or plasmenyl) substituent. The suffix, e, indicates the presence of an ether linked substituent, although plasmanyl or plasmenyl classification has not been confirmed. An "x" in the name of some NEFA, FAA and NAE indicates the unknown position the double bounds, i.e., 18:1n-x for FFA09. Most of the oxFA are not identified, but sub-classified as Hydroxy-eicosatetraenoic acids, Epoxy-eicosatetraenoic acids, Hydroxy-octadecadenoic acids, Oxo-octadecadenoic acids or Dihydroxyeicosatrienoic acids.
